# Profiling metabolome of mouse embryonic cerebrospinal fluid following maternal immune activation

**DOI:** 10.1016/j.jbc.2024.107749

**Published:** 2024-09-07

**Authors:** Boryana Petrova, Tiara E. Lacey, Andrew J. Culhane, Jin Cui, Jeannette R. Brook, Alexander Raskind, Aditya Misra, Maria K. Lehtinen, Naama Kanarek

**Affiliations:** 1 Department of Pathology, Boston Children's Hospital, Boston, Massachusetts, USA; 2 Harvard Medical School, Boston, Massachusetts, USA; 3 Graduate Program in Biological and Biomedical Sciences, Harvard Medical School, Boston, Massachusetts, USA; 4 IROA Technologies^TM^ LLC, Chapel Hill, North Carolina, USA; 5 Harvard-MIT Division of Health Sciences and Technology, Massachusetts Institute of Technology, Cambridge, Massachusetts, USA; 6 Broad Institute of Harvard and Massachusetts Institute of Technology, Cambridge, Massachusetts, USA

**Keywords:** embryonic cerebrospinal fluid, metabolic composition of the embryonic cerebrospinal fluid, developmental metabolomics, maternal immune activation, embryonic metabolism

## Abstract

The embryonic cerebrospinal fluid (eCSF) plays an essential role in the development of the central nervous system (CNS), influencing processes from neurogenesis to lifelong cognitive functions. An important process affecting eCSF composition is inflammation. Inflammation during development can be studied using the maternal immune activation (MIA) mouse model, which displays altered cytokine eCSF composition and mimics neurodevelopmental disorders including autism spectrum disorder (ASD). The limited nature of eCSF as a biosample restricts its research and has hindered our understanding of the eCSF’s role in brain pathologies. Specifically, investigation of the small molecule composition of the eCSF is lacking, leaving this aspect of eCSF composition under-studied. We report here the eCSF metabolome as a resource for investigating developmental neuropathologies from a metabolic perspective. Our reference metabolome includes comprehensive MS^1^ and MS^2^ datasets and evaluates two mouse strains (CD-1 and C57Bl/6) and two developmental time points (E12.5 and E14.5). We illustrate the reference metabolome’s utility by using untargeted metabolomics to identify eCSF-specific compositional changes following MIA. We uncover MIA-relevant metabolic pathways as differentially abundant in eCSF and validate changes in glucocorticoid and kynurenine pathways through targeted metabolomics. Our resource can guide future studies into the causes of MIA neuropathology and the impact of eCSF composition on brain development.

The cerebrospinal fluid (CSF) provides a protective fluid cushion for the brain (1, 2) and delivers neuroactive proteins, peptides, and small molecules that are critical for normal brain development (3, 4). Neural progenitor cells of the developing cerebral cortex are in direct contact with the CSF, and the CSF composition of growth factors and nutrients is critical for their healthy development, differentiation, and proliferation (5, 6, 7) underscoring the importance of the metabolic content of the CSF for normal development. The CSF composition also informs metabolic state and brain development stages (5, 8), and CSF is sampled for biomarkers of infections and neurologic diseases (3, 4) including the elucidation of the transcriptome, proteome, and some diagnostic biomarkers in the CSF during development (3). However, while meaningful efforts have characterized some small molecules in the CSF as critical for its function, *i.e.*, nutrients, ions, and signaling molecules, the CSF metabolome remains relatively less characterized.

To date, efforts to establish a reference (normative) adult mouse CSF metabolome (9, 10, 11) have identified a growing list of CSF metabolites associated with adult neuroinflammation as well as neurologic and psychiatric phenotypes (12). However, the CSF metabolome during development in mouse models of human-relevant pathology has yet to be comprehensively described. This gap in knowledge and research resources compromises our understanding of normal development as well as the etiology of neurodevelopmental disorders.

Here we describe the profiling of the embryonic CSF (eCSF) metabolome that is of interest to developmental biologists and neuroscientists and share this resource with the community. Utilizing liquid chromatography-mass spectrometry (LC-MS), we generated a comprehensive database of embryonic day (E)14.5 mouse eCSF of CD-1 mice, including mass verification at the level of mass-to-charge (MS^1^) or the level of fragmentation spectrum (MS^2^). To exemplify the utilization of this database as a reference library for future studies we implemented it in metabolite profiling of healthy eCSF from two mouse strains (C57BL/6 and CD-1) and two developmental time points (E12.5 and E14.5). We also used the reference library for metabolite profiling of eCSF from embryos that were exposed to maternal immune activation (MIA) by polyinosinic-polycytidylic acid (polyI:C, a synthetic viral mimetic) injection of pregnant dams. We chose this model of inflammation-induced neuropathology because a growing body of evidence suggests that changes in eCSF composition following maternal illness are likely to contribute to negative sequelae in offspring (13, 14, 15, 16), such as neurocognitive impairment, schizophrenia, and autism spectrum disorders (ASD) (17, 18, 19, 20, 21). In eCSF from the MIA model, we observed a differential abundance of metabolites from two metabolic pathways related to inflammation (22): glucocorticoids (23) and the kynurenine pathway (24, 25, 26).

The choice of time points and mouse strains were led by relevance to the MIA mouse model: The E12.5 developmental timepoint coincides with the peak levels of pro-inflammatory cytokines in maternal plasma 3 h following MIA induction by exposure to polyI:C (27). The E14.5 developmental timepoint coincides with reported abnormalities following MIA induction of brain cytokine levels including IL-6 (28, 29, 30, 31) and IL-17a (32, 33, 34). Additionally, we previously reported a proinflammatory eCSF state at E14.5 that includes elevated levels of CCL2 in the eCSF that is accompanied by the accumulation of immune cells (35).

Our eCSF metabolomics database provides an important resource for guiding investigations of neural development. Additionally, we uncovered several metabolic aberrations induced by MIA, including increased levels of metabolites of rodent corticoid stress response and the kynurenine pathway across the maternal-fetal axis. Our work may reveal targetable pathways for developing CSF-based interventions for at-risk offspring following maternal illness.

## Results

### Generation of an embryonic CSF metabolome database

We applied untargeted metabolomics to generate a library of polar metabolites in mouse eCSF at embryonic day (E)14.5. To ensure the purity of the embryonic CSF and consistency amongst independent experiments, we followed established procedures (6), including, when feasible, evaluation for blood contamination by comparing the relative abundance of the intracellular metabolite glucose-6-phosphate (G6P), that is present in plasma or serum samples due to leakage from the lysis-prone red blood cells but is not expected to be detected in CSF. We noted a strong depletion of this metabolite from eCSF (Fig. S1*A*), confirming the purity of the analyzed eCSF samples. Of note, although sex differences are noted in adult CSF (36), where 15 to 20 μL of CSF can be collected and analyzed per animal, analyzing eCSF with LC-MS requires pooling samples from all the embryos in an entire litter to obtain a volume of 10 to 15 μL. Altogether, this technical limitation prevented stratification by sex.

In addition, due to the high sensitivity of mass spectrometry, background and contaminant signals with defined m/z values and retention times are abundant and can sometimes be indistinguishable from true biological signals (37). Signals detected by the mass spectrometer are called “features” and these include biological analytes, contaminants, and technical noise. This noise becomes especially confounding when the sample, like CSF, contains a low abundance of metabolites. Therefore, a rigorous analytical method is required to ensure that our metabolite library construction is based on true biological signals from eCSF.

**Figure 1:**
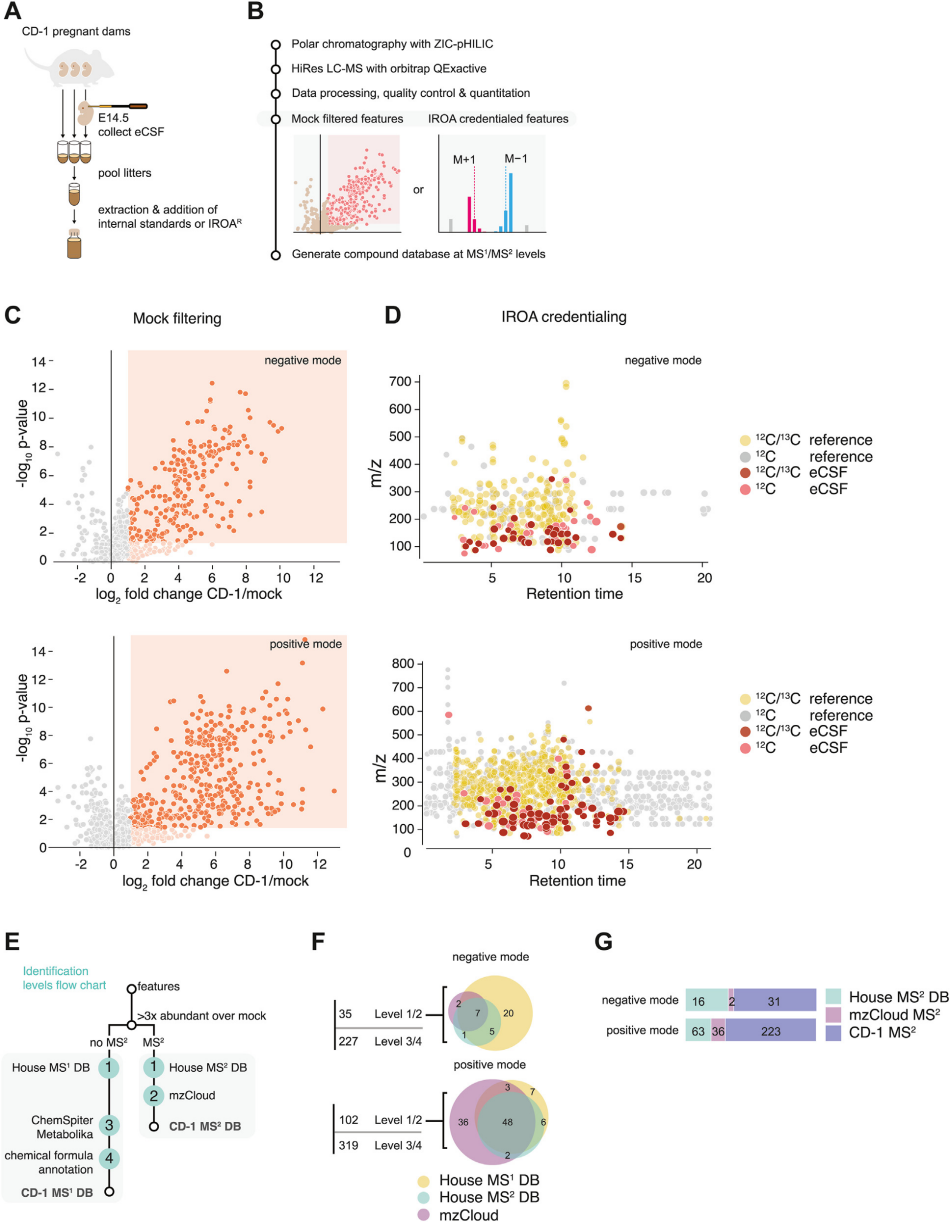
**An approach to generate an embryonic CSF (eCSF) compound database as a tool for future developmental and neurological research.***A*, schematic depicting experimental workflow for embryonic CSF sample preparation. CSF from embryos (eCSF) from a single pregnant mouse was pooled and 3 to 7 μl extracted for analysis. Each pregnant mouse is considered a biological replicate. *B*, schematic depicting the analytical workflow for the generation of an eCSF polar compound database. Polar chromatography was performed in HILIC mode (ZIC-HILIC: zwitterionic hydrophilic interaction column) and metabolites were detected using a high-resolution (HiRes) mass spectrometer (Thermo Orbitrap QEactive). Data was processed using two separate approaches depending on the compound identification strategy. Contaminants were filtered by either “Mock filtering” - based on an extensive set of mock samples, or by “IROA” credentialing – based on labeled metabolome correspondence (Fig. S1, *B*–*C*). Signals (features) that passed the abundance filter (mock filtered features) and quality control were then used to generate compound databases at MS^1^ and MS^2^ levels. *C*, Representative results from untargeted polar metabolomics on eCSF from CD-1 mice (n = 7) for mock-filtered dataset outlined in (*B*). Negative- and positive-mode analyses are shown separately. Mock and eCSF samples are compared using a volcano plot. The signal was normalized based on targeted analysis of polar metabolites and mean-centering per sample (see methods for details). Metabolites highlighted in *orange* are >2-fold significantly higher in eCSF than in mock samples. *D*, representative results from untargeted polar metabolomics of eCSF from CD-1 mice (n = 7) for IROA credentialed dataset as outlined in B. Relevant biological features were identified using IROA-“LTRS” – a 1/1 mix of 95:5/5:95 unlabeled (^12^C) and ^13^C-labeled reference yeast metabolome (^12^ C/^13^C reference, IROA TruQuant Yeast Extract Semi-targeted QC Workflow). IROA-credentialed features were identified from eCSF (^12^C, unlabeled) mixed with a reference internal standard (“IS”) that was composed of 5:95 unlabeled (^12^C) and ^13^C-labeled metabolome. Non-credentialed ^12^C signals (^12^C reference or ^12^C eCSF), with no matching IROA ^13^C signals, are depicted as indicated in the legend. *E*, Strategy for the implementation of the eCSF library into an untargeted analysis workflow combining in-house and online databases at MS^1^ and MS^2^ levels. Levels of identification certainty (1 through 4) are depicted following recommendations from the metabolomics community. *F*, breakdown of the composition of the eCSF-specific filtered features from the mock-filtered dataset. Negative- and positive-mode are presented separately. The *left* panels present the fraction of total filtered features at different identification levels (with or without database matches). *Right* panels (colored Venn diagrams) represent the breakdown of database-matched features identified either at Level 1 (exact mass, retention time, and MS^2^ match or exact mass and RT match only) or Level 2 (exact mass and external database MS^2^ match only). *G*, Breakdown of the composition of the eCSF-specific filtered features from the mock-filtered CD-1 untargeted dataset for which we have obtained MS^2^ level information. Negative and positive mode are presented separately. Matches to in-house (House MS^2^ DB) and external (mzCloud MS^2^) libraries are depicted. The remaining spectra were manually quality controlled and consolidated into the CD-1 MS^2^ database.

To that end, we compared the ability of two analytical approaches to identify non-biological background (noise): 1) mock filtering and 2) “IROA” (Isotopic Ratio Outlier Analysis, Fig. 1, *A* and *B*). In the “mock filtering” approach, we compared the eCSF profile to multiple mock samples. The mock samples were empty tubes that underwent the exact same series of sample preparation steps through injection into the LC-MS, and thus they only differed in the actual presence of eCSF in the collection tubes. This approach exposed background features that were shared by the mock samples and eCSF samples, including technical noise and signals of non-biological contaminants. These signals were excluded from our defined eCSF metabolome. True eCSF-specific signals were defined as signals with 3-fold higher abundance in eCSF compared to mock samples (Fig. 1*C*). In the “IROA” approach (38), we employed a ^13^C-labeled reference metabolome to credential detected signals (Fig. S1, *B* and *C*). Briefly, in this approach, we first mixed the eCSF metabolome with a ^13^C-labeled reference metabolome, a metabolome where most naturally occurring ^12^C carbon atoms have been substituted with ^13^C, a heavier stable isotope of carbon. Because LC-MS resolution allows detection of the common ^12^C-metabolites side-by-side with the reference ^13^C metabolites, detected signals in the eCSF could be credentialed as “true biological signals” only if they had a matched heavy signal in the reference metabolome. All other signals were excluded from the analysis (Fig. 1*D*). In LC-MS, to enable detection on the MS analyzer, metabolites are ionized by either gaining a proton in positive mode or losing one in negative mode. Thus, we analyzed metabolites in both modes for comprehensive assessment in both “mock filtering” and “IROA” approaches.

Both the mock filtering and IROA approach excluded a similar order of magnitude number of signals: mock filtering retained 16% (negative mode) and 18% (positive mode) of total features after mock filtering (Fig. 1*C*) while IROA-based filtering retained 12% (negative mode) and 16% (positive mode) (Fig. 1*D*). Overall, we detected more signals using the mock filtering approach. We detected 632 (negative mode) and 798 (positive mode) features compared to the 35 (negative mode) and 71 (positive mode) detected using IROA. We concluded that the IROA approach is too stringent for eCSF metabolite profiling, likely due to the use of a yeast metabolome IROA library rather than a CSF-specific labeled metabolome, which is not currently available. Therefore, we proceeded to apply the mock filtering approach for all subsequent analyses.

For careful annotation and identification of detected metabolites, we employed a scoring system based on community recommendations for best practices in reporting untargeted metabolomics datasets (39, 40, 41) (Fig. 1*E*). The scoring system was applied to features detected in eCSF in both negative and positive modes (Fig. 1*F*). We incorporated into our workflow extensive in-house standard reference libraries, both at MS^1^ and MS^2^ levels, as well as information from several online databases. Level 1 reflects the highest level of certainty, and it integrates chemical information from retention time matching with MS^1^ (high-resolution exact mass) and/or MS^2^ data from in-house standards reference libraries that are chromatographic-method specific. At this level of identification, if a detected feature matched a standard metabolite from our MS^1^ in-house libraries, we denoted it as a “House_MS^1^_DB”, whereas if it matched MS^2^ in-house libraries, we denoted it as “House_MS^2^_DB” (Fig. 1*F*). For Level 2 annotation, we relied on the mzCloud online spectral database as our reference, and because no specific chromatographic method retention times are available when using mzCloud, this level reflects lower certainty in compound identification. Yet, the utilization of mzCloud extended our compound annotation and added 2 metabolites in negative mode and 36 metabolites in positive mode to our defined eCSF metabolome (Fig. 1*F*). Finally, all remaining compounds for which we were able to obtain a chemical formula (227 in the negative mode and 319 in the positive mode) were annotated at certainty Levels 3 and 4 and given molecular weight and retention time labels. In cases when a chemical formula matched entries from external compound databases (Metabolika or ChemSpider), the name of the top listed hit from the database was used and the level of certainty was depicted as Level 3 (putative annotation). For many metabolites that were scored as Level 3, we also obtained MS^2^ data and labeled the dataset of MS^2^-verified metabolites “CD-1 MS^2^” (31 of 49 in negative mode and 223 of 322 in positive mode, Fig. 1*G*). This could be extremely helpful with metabolite identification in future studies using our spectral database. All detected signals with insufficient intensity to assign a molecular formula were excluded from our database. Although we could not verify the identity of all annotated signals (below Level 1), our data suggest that these are eCSF-specific, relevant metabolites and not contaminants. However, we cannot exclude the possibility that some are artifacts from oxidation, chromatography, and ionization; these signals could be degradation products or chemical derivatives of other compounds. Therefore, care must be taken in interpreting results, and extensive follow up experiments will be required to fully annotate the eCSF-specific metabolome. In sum, as a resource for future studies, we report here a stringent list of metabolites detected in the mouse eCSF at day E14.5, with assigned annotation, and a transparent report of annotation confidence.

### Application of the reference library for comparative untargeted eCSF metabolomics in two mouse strains and at two developmental time points

The metabolic profile (42), and behavioral and immunological effects of inflammation are strain-dependent (43, 44). We therefore tested the applicability of our CD-1 mouse eCSF metabolomics database in C57Bl/6 mice. Like C57Bl/6 mice, CD-1 mice can also be used to model MIA (33, 35). Thus, we provide extensive reference data for future studies of eCSF of both CD-1 and C57Bl/6 mouse strains.

**Figure 2:**
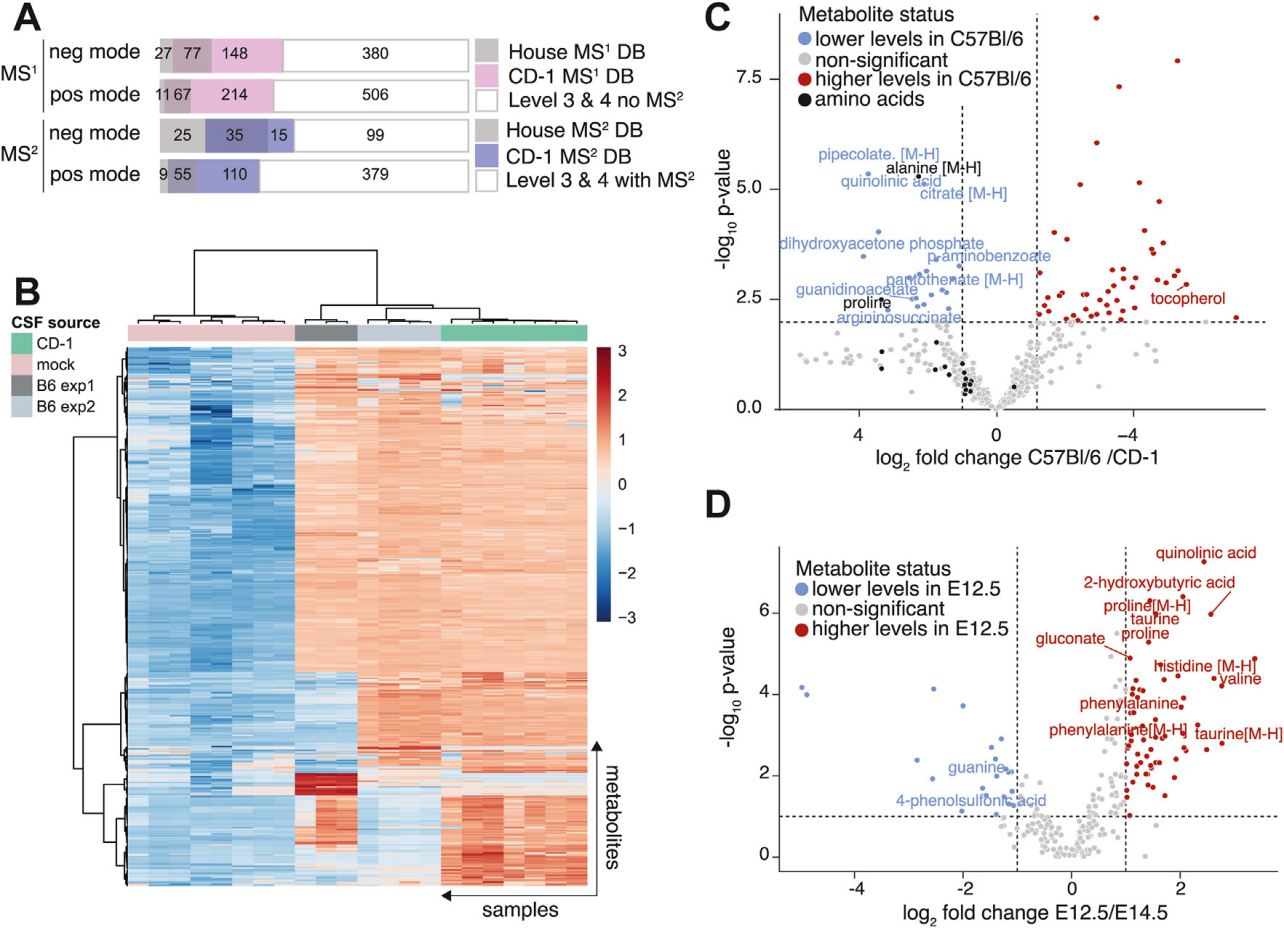
**Application of eCSF library in untargeted eCSF metabolomics in two mouse strains and at two developmental time points.***A*, breakdown of the composition of the eCSF-specific filtered features based on overlapping features between CD-1 and C57Bl/6 eCSF untargeted metabolome at E14.5. Two independent C57Bl/6 eCSF cohorts and one CD-1 cohort were treated as distinct batches because sample collection was performed on different days (minimum of three biological replicates per cohort). Filtered untargeted features from C57Bl/6 with either an in-house database match (House MS^1^ DB) or a CD-1 database match (CD-1 MS^1^ DB) are shown for MS^1^ or MS^2^ databases as indicated. The remaining features, with or without MS^2^-level information, which were not present in our databases, were identified at Level 3 and 4 and are also indicated. *B*, heatmap for the combined positive- and negative-mode data in (*A*). Mock samples were included for comparison. Further 5% (interquartile range) filtering step, log-transformation and Pareto scaling were performed within the MetaboAnalyst platform. *C*, Volcano plot for the combined positive and negative mode data in (*A*). For clarity, only significantly changed metabolites between CD-1 and C57Bl/6 eCSF with in-house library match were annotated, and negative ion adducts were further specified. In addition, all detected amino acids are indicated in black, with only significantly altered (alanine and proline) annotated. *D*, Volcano plot, depicting a comparison between polar metabolites detected by untargeted metabolomics from eCSF (C57Bl/6 samples) of E12.5 and E14.5. Compounds from Levels 1 to 3 were used to generate the plot. For clarity, only significantly changed metabolites with in-house library match are annotated. The label [M-H] depicts ions that were detected in negative mode.

We used our curated CD-1-eCSF database (CD-1-DB, Fig. 1, labeled “CD-1”) and our in-house compound databases (labeled “in-house”) as reference libraries for both CD-1 and C57Bl/6 eCSF samples at MS^1^ and MS^2^ levels. More features matched to CD-1 library than to the in-house library and there was a good overlap between the two (Table 1 and Fig. 2*A*).

**Table 1:** Number of features from CD-1 and C57Bl/6 eCSF samples identified as matching features to the reference metabolite libraries “CD-1” and “in-house”

Ionization mode	CD-1 library	In-house library	Double match (both libraries)
MS^1^			
Negative mode	148	27	77
Positive mode	214	11	67
MS^2^			
Negative mode	15	25	35
Positive mode	110	9	55

To address the question of overlap between the two different mouse strains, we compared the total number of filtered features to the total features in the CD-1-DB. We detected 270 negative mode metabolites in the C57Bl/6 eCSF metabolome, out of the 421 metabolites present in our negative mode CD-1-DB (64%). Similarly, we detected 198 positive mode metabolites in the C57Bl/6 eCSF metabolome, out of the 262 metabolites present in the positive mode CD-1-DB (76%). We assumed that at least some of the metabolites that were detected in eCSF from CD-1 mice but not in C57Bl/6 mice could be missing from the C57Bl/6 samples for technical, and not biological reasons (*e.g.* LC-MS related retention time shifts or batch effect-related changes in sensitivity of detection). This is also true for differences between the two independent experiments we performed in C57Bl/6 mice (Fig. 2*B*, and S2, *A* and *B*). We applied a highly stringent filter in our analysis that is based on a comparison of the samples to the integrated sum of false signals from an extensive number of mock samples. We applied this stringent filter because we prioritized the reduction of false positive results over false negative results. Therefore, this step likely resulted in the exclusion of metabolites that varied between the two mouse strains possibly due to batch effects. Nevertheless, biological reasons could dictate dissimilar gene expression and metabolism both in the mothers and in the embryos in these two strains. The hundreds of metabolites that were detected in both mouse strains allowed us to report in more detail the similarities and differences between the CD-1 and C57Bl/6 eCSF metabolomes (Figs. 2*B*, S2, *A* and *B*). Abundant Level 1-verified metabolites, which included most amino acids, exhibited no significant relative differences between the two strains examined (Fig. 2*C*). Among the significantly different metabolites were Level 3 and 4 putatively annotated compounds that were present in our CD-1-DB but not in our in-house or online databases. These metabolites that we annotated and scored in our eCSF database represent potentially novel compounds that could introduce interesting metabolic differences to be considered in future eCSF research.

Next, we focused on C57Bl/6 mice and applied our eCSF database to assist in untargeted metabolomics characterization of eCSF from two embryonic stages relevant for the MIA model, E12.5 and E14.5. Collection of eCSF from these two developmental stages was performed and run on our instruments on different days, which may cause experimental variability due to batch effects. Therefore, to minimize further technical variability, we integrated data from two independent experiments (each with eCSF collected from a minimum of three independent litters). We detected a differential abundance of several metabolites between E12.5 and E14.5 (Fig. 2*D*), including the amino acids taurine, histidine, proline, phenylalanine, and valine. These amino acids were abundantly detected, and their differential levels reflect metabolic changes that occur over the course of brain development. Amino acids that were reported to be significantly altered in the fetal brain across development and during hyperglycemia in maternal diabetes, such as aspartate, asparagine, glutamate, and GABA (45), were not different in the eCSF in E12.5 vs. E14.5. These results reflect the distinct metabolic compositions of brain tissue and the CSF, underscoring the unique metabolic composition of the eCSF that we explored here, and the importance of including the unique CSF metabolome rather than relying on brain parenchymal changes to infer CSF values. The dataset presented here can further serve the community as a reference for eCSF-specific metabolites and inform on metabolic alterations during these critical stages of embryonic development.

### Application of eCSF reference metabolome library in eCSF untargeted metabolomics study following MIA

**Figure 3:**
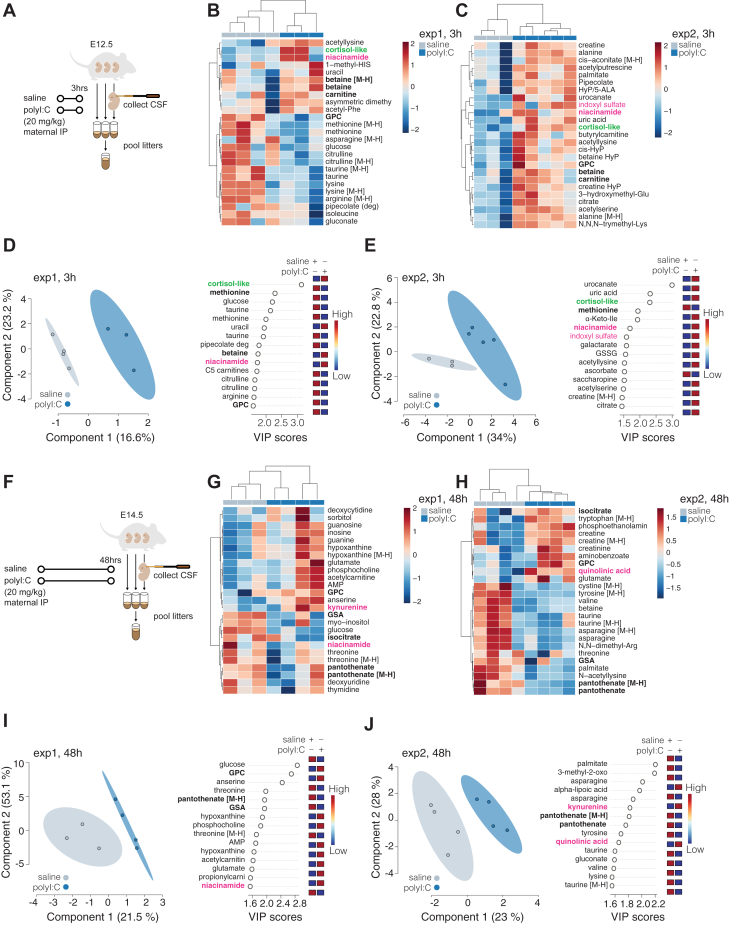
**Application of eCSF library in the study of eCSF in a maternal immune activation (MIA) model.***A*, schematic depicting the collection strategy of eCSF for untargeted LC-MS metabolomics 3 h post induction of MIA by injection with polyI:C. *B–E*, altered metabolites 3 h post polyI:C injection. Shown are heatmaps of top 25 changed metabolites (*B* and *C*) or PLSDA and corresponding important features (VIP) plots (*D* and *E*) of untargeted metabolomics analysis of polar eCSF metabolites from treatment and control samples (minimum of three biological replicates per condition). Two independent experiments are presented separately (*B* vs *C* and *D* vs *E*). Metabolites of interest are highlighted in *green* (glucocorticoid pathway), and *magenta* (kynurenine pathway). Metabolites overlapping between the top 25 analysis and VIP analysis are in bold. Plots were generated using the online MetaboAnalyst tool, after log-transformation and Pareto scaling of the combined data from normalized negative and positive modes analyses. Level 1 (exact mass, RT match or RT and MS^2^ match to in-house databases) confidence metabolites were used for this analysis. For a comparison between saline and polyI:C conditions based on Level 1 to 4 metabolites (see Fig. S3, *E* and *F*)*.F*, schematic depicting the collection strategy of eCSF for untargeted LC-MS metabolomics 48 h post-induction of MIA by injection with polyI:C. *G*–*J* Same as for (*B*–*E*) except data is from samples collected 48 h post-induction of MIA. (deg), fragment; HyP, hydroxyproline; GCP, glycerophosphocholine; GSA, guanidinosuccinate; [M-H], ion detected in negative mode.

To comprehensively profile metabolic changes in eCSF following induction of inflammation in the mother, we collected eCSF 3 h and 48 h following polyI:C delivery (Fig. 3, *A* and *F*). We confirmed elevated cytokine levels in serum from polyI:C exposed dams at 3 h (Fig. S3, *A* and *B*) and near baseline levels in serum by 48 h following polyI:C exposure (Fig. S3, *C* and *D*). We independently processed eCSF samples from two independent experiments per time point and analyzed them by untargeted metabolomics. We detected significant changes among eCSF metabolites between embryos collected from saline-*versus* polyI:C-injected dams at both time points (Fig. S3, *E* and *F*). We observed significant biological variability between our independent experiments, as has been reported for the maternal response to polyI:C previously (44, 46), and therefore focused on pathways that consistently changed across the two experiments and experimental conditions. At 3 h following polyI:C delivery, we noted a consistent change in cortisol-related metabolites (Fig. 3, *B*–*E*). While, at 3 h and at 48 h we observed several changes in the kynurenine pathway (Fig. 3, *B*, *C*, *G*, *H*, *I* and *J*) including the metabolites indoxyl sulfate, kynurenine, quinolinic acid and niacinamide. The changes in metabolites of the kynurenine pathway were consistent with previous reports of association between inflammation and elevated kynurenine in embryonic mouse brains (24, 47). Further, these kynurenine pathway alterations in eCSF also coincide with a pro-inflammatory eCSF cytokine profile observed previously upon polyI:C injection at the equivalent time point (35). In summary, the application of our eCSF metabolome library to untargeted metabolomics enabled the discovery of altered metabolite levels in key pathways related to MIA model pathology—specifically, corticosteroid and kynurenine metabolism.

### Targeted metabolomics for validation of the MIA-induced changes in corticosteroid and kynurenine pathways in eCSF

**Figure 4:**
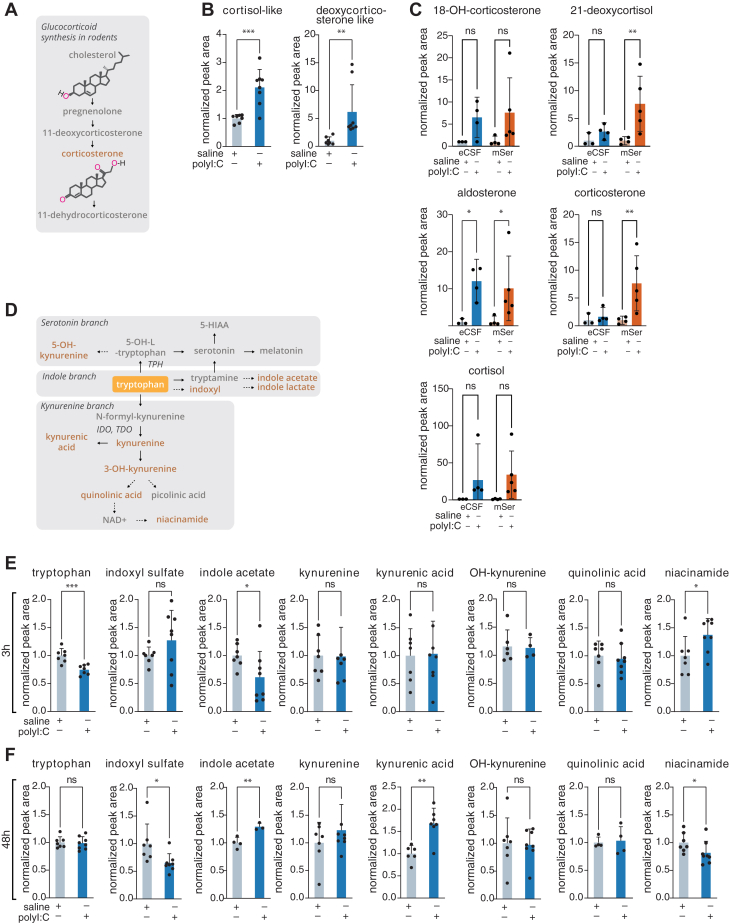
**Targeted metabolomics of MIA eCSF reveals involvement of kynurenine pathway and a corticoid stress response.***A*, schematic of glucocorticoid synthesis in rodents. In orange – corticosterone, was detected by targeted metabolomics in MIA eCSF. *B*, Bar graphs depicting mean ± standard deviation of two corticosteroids identified at Level 2 confidence (including m/z match to the online database with no RT) by untargeted metabolomics 3 h post polyI:C injection. Two independent experiments (each with a minimum of three biological replicates) were combined for the analysis (leading to a minimum of 7 biological replicates per condition). Missing values for individual metabolites (in cases where measurements were under our limit of detection) were omitted from the analysis. ∗∗ = *p* < 0.001; ∗∗∗ = *p* < 0.001 (Unpaired *t* test with Welch's correction). *C*, Bar graphs depicting mean and standard deviation levels post polyI:C injection from eCSF or maternal serum (mSer) for different glucocorticoid-related metabolites detected by our LC-MS ∗ = *p* < 0.05; ∗∗ = *p* < 0.005 (one-way ANOVA, Šídák's multiple comparisons test). Graphs are presented relative to eCSF abundance in control. “OH” denotes “hydroxy”. *D*, schematic of tryptophan degradation pathways in mammals. Metabolites present in our in-house library and analyzed *via* targeted metabolomics are highlighted in *orange*. “OH” denotes “hydroxy”. *E* and *F*, Mean and standard deviation of indicated metabolites 3 h (*D*) or 48 h (*E*) post polyI:C injection. Data were analyzed using targeted metabolomics approach. Two independent experiments were combined for each time point. 3h data includes exp 1 and 2: exp 1 with n = 3 for saline group and n = 4 for polyI:C group, and exp 2 with n = 4 in each group. 48h data includes exp 1 and 2: exp 1 with n = 4 for saline group and n = 3 for polyI:C group, and exp 2 with n = 3 in the saline group and n = 5 in the polyI:C group. ∗ = *p* < 0.01; ∗∗ = *p* < 0.001; ∗∗∗ = *p* < 0.001 (unpaired *t* test, two-tailed). Of note, our method could not differentiate 5- and 3-hydroxykynurenine, labeled here simply as “OH-kynurenine”.

We next applied targeted metabolomics of eCSF to validate and further investigate the elevated levels of glucocorticoids and kynurenine pathway components we observed (Fig. 4, *A* and *D*). We first used the combined eCSF data from two independent experiments designated Experiments 1 and 2 (see Fig. 3) at 3 h following polyI:C exposure and focused on two molecules (Fig. 4*B*): a molecule at m/z 363.2166 (that we putatively identified as cortisol based on its mass and labeled as “cortisol-like”) and a molecule at m/z 344.1985 (putatively identified and labeled “deoxycorticosterone-like”). These metabolites respectively increased approximately 2- to 5-fold in eCSF at 3 h following polyI:C compared to saline control. We developed an LC-MS method suitable for sterol separation (Fig. S4*A*) and highly sensitive detection (Fig. S4*B*). Out of the nine tested known sterols, five were detected in both eCSF and maternal serum (Fig. 4*C*, and S4*C*). Levels of several sterols in polyI:C treated samples were significantly higher than in control (Fig. 4*C*). However, considering some experimental limitations, such as long-term storage and additional freeze-thaw cycles of these samples, that were shown to affect labile metabolites in eCSF samples (36), there is a risk of technical interference with precise interpretation especially for eCSF, where we observed that levels of sterols were especially low compared to maternal serum (Fig. S4*C*). These limitations do not apply to the sterols we observed in our untargeted analysis (Fig. 4*B*), which was performed first. A clear chromatographic signal was observed for both cortisol-like and deoxycorticosterone-like (Fig. S5*A*). In fact, both signals overlapped with cortisol’s retention time. We were unable to obtain MS^2^ level information for these signals but based on the exact mass and retention time match to chemical standards, we suggest that these signals are cortisol [M-H]^-^ and an ionization adduct or derivative of cortisol [M-H_2_O-H]^-^. Indeed, although considered a much less abundant sterol in mice, cortisol has been previously observed in mouse serum (48). Neither one of these molecules was present in eCSF at 48 h, suggesting that these changes in the eCSF metabolome track with the fast clearance of maternal serum cytokines (Fig. S3*D*), and that the glucocorticoid aspect of the maternal inflammatory response transmitted to the embryo was largely resolved by 48 h. These results suggest modulation of corticosterone synthesis upon inflammation. Further investigation is required for the study of the biological role of diverse corticosterone metabolites in neuropathology in the MIA model.

Next, we applied metabolite annotation verification of the kynurenine pathway (Fig. 4*D*) using standards (Fig. 4, *E* and *F*). Tryptophan levels were reduced while niacinamide levels were increased at 3 h (Fig. 4*E*). At 48 h, we observed increased kynurenic acid and indole acetate levels, while niacinamide and indoxyl sulfate levels decreased (Fig. 4*F*). Of note, kynurenine and quinolinic acid, which were significantly elevated in the polyI:C exposed embryos in our untargeted analysis of individual Experiments 1 and 2 (Fig. 3), were not significantly higher when both experiments were combined. We optimized a separate method for detection of serotonin, picolinic acid, and 5-HIAA standards, but were unable to consistently detect these metabolites in 3 to 5 μl samples of eCSF, likely due to low abundance or low ionizing efficiency on the MS. These results demonstrate changes in different kynurenine pathway metabolites at the two time points analyzed and suggest two previously reported tryptophan utilization pathways *via* kynurenine or indoxyl (Fig. 4*D*) at 3 h and 48 h following polyI:C delivery.

### Study clearance of kynurenine pathway intermediates in the maternal-embryonic axis

Our data raise the question of whether the elevated kynurenine is produced locally in the embryo or originates from the pregnant dam, as kynurenine can cross the placenta (49). Further, Bonnin and colleagues found that maternal tryptophan is metabolized into 5-HT in the placenta and then utilized in the fetal forebrain (50), which raises the possibility that kynurenine in the eCSF could be exogenously produced in the placenta (49). The source of the kynurenine is likely to have implications on the dynamics of the accumulation and clearance of kynurenine and kynurenine-related metabolites. To address a possible maternal contribution to the elevation of kynurenine metabolites in the eCSF, and to test the dynamics of kynurenine trafficking and clearance, we next tested the effects of kynurenine supplementation to mothers (Fig. S6*A*). Pregnant dams received intraperitoneal (IP) injections of either saline or kynurenine (20 mg/kg). Maternal serum (mSer), embryonic serum (eSer), and eCSF were subsequently collected at different time points. Due to the inherent nature of sample collection and low volumes of fluids available at these early ages, samples were collected across different days, and replicates were obtained from independent mice. Thus, we attribute some experimental variability to technical batch differences. Nevertheless, while tryptophan levels remained unchanged (Fig. S6*B*), kynurenine levels increased in maternal serum following kynurenine supplementation and were re-normalized approximately 90 min later (Fig. S6*C*). To test the dynamics and distribution of kynurenine and other kynurenine pathway intermediates in the eSer and eCSF, we modeled the occurring dynamic changes assuming a linear geometric model of metabolite utilization and/or clearance (Fig. S6, *C*–*E*). Kynurenine clearance or utilization seemed to follow similar dynamics in mSer, eSer, and eCSF. Thus, higher levels of kynurenine in mSer were dynamically and rapidly reflected in the levels of kynurenine in eSer and eCSF, suggesting kynurenine can quickly redistribute from the maternal serum to eCSF. Similarly, kynurenic acid redistribution was not significantly different between the three biofluids but there was a trend towards a slower decay in kynurenic acid levels in eCSF, suggesting delayed clearance or utilization (Fig. S6*D*). We further compared the dynamic change of kynurenine, kynurenic acid, and quinolinic acid within the eCSF (Fig. S6*E*). Here, we observed significantly different dynamics of quinolinic acid relative to kynurenine, which could be explained by a delay in the synthesis, utilization, or distribution of this metabolite in eCSF or the existence of an orthogonal contribution. These observations might reflect the dynamics of resolution of inflammation in eCSF and potentially explain why we see certain metabolic alterations at 48 h but not at 3 h post polyI:C injection in our MIA model. However, future experiments utilizing metabolic labeling are required to expand on this finding, to distinguish between exogenous and endogenous sources of kynurenine and its intermediates, and to shed light on redistribution vs. embryonic synthesis of these metabolites.

Together, these results exemplify the application of our eCSF metabolome reference library for the study of eCSF. The study revealed MIA-induced changes in two metabolic pathways, corticosteroid metabolism and the kynurenine pathway. Future studies can further utilize our eCSF reference library to investigate whether changes in the levels of these metabolites in the eCSF following MIA mediate, compensate, or simply reflect the severity of MIA and/or MIA-induced pathology in the embryonic brain.

### Differential dynamics of glucocorticoid and kynurenine pathway intermediates in the maternal-embryonic axis

**Figure 5:**
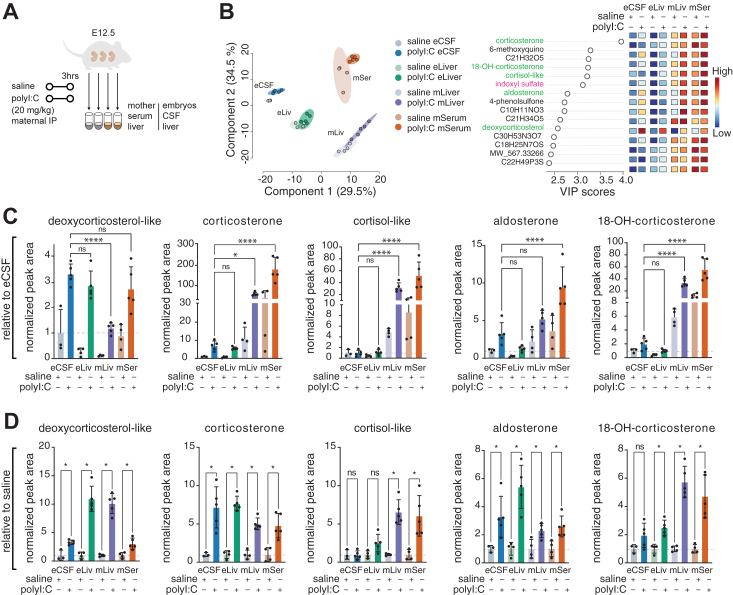
**Metabolic changes in glucocorticoid-related intermediates as assessed by untargeted metabolomics in embryonic liver and CSF or maternal liver and serum.***A*, schematic depicting the collection strategy of embryonic CSF (eCSF) and liver (eLiv) and maternal liver (mLiv) and serum (mSer) for untargeted LC-MS metabolomics 3 h post-induction of MIA by injection with polyI:C. *B*, PLSDA and corresponding important features plot of untargeted metabolomics analysis of polar eCSF, eLiv, mLiv, and mSer metabolites from polyI:C- and saline-treated samples at 3 h post injection (each with a minimum of three biological replicates). Plots were generated using the online MetaboAnalyst tool, after log-transformation and Pareto scaling of the combined data from normalized negative- and positive-mode analysis. Levels 1 to 3 (exact mass, RT match, MS^2^ match to in-house or online databases, and match to eCSF CD-1 library) confidence metabolites were used for this analysis. eCSF data from experiment 2 from previous figures is from the same mouse cohort and was re-analyzed for this set of graphs. Metabolites of interest are highlighted in *green* (glucocorticoid pathway) and *magenta* (kynurenine pathway). “OH” denotes “hydroxy”. *C* and *D*, Bar graphs depicting mean and standard deviation levels of indicated metabolites post polyI:C injection for different glucocorticoid-related metabolites detected by our LC-MS ∗ = *p* < 0.01; ∗∗ = *p* < 0.001; ∗∗∗ = *p* < 0.001; ∗∗∗∗ = *p* < 0.0001 (one-way ANOVA, Šídák's multiple comparisons test). Graphs are either presented relative to eCSF abundance (*C*) or relative to the corresponding control saline sample set (*D*). “OH” denotes “hydroxy” CD.

MIA imparts systemic embryonic stress, with impact on the brain and the hematopoietic system (51, 52). At both E12.5 and E14.5, the developing embryonic hematopoietic system is located in the liver (53). We therefore profiled the metabolic state of the glucocorticoid and kynurenine pathways in the embryonic liver. Of note, embryonic livers were pooled from the entire litter due to their small size, allowing for consistent metabolic profiling. Additionally, we analyzed the maternal liver because the adult liver expresses relevant metabolic enzymes in both the glucocorticoid and the kynurenine pathways (54). We found differential kinetics of key metabolites between maternal and embryonic biofluids upon kynurenine injection (Fig. S6), so we performed a side-by-side analysis between maternal serum (mSer), maternal and embryonic livers (mLiv, eLiv), and eCSF. Our data indicated that fast metabolism occurs within hours in both mothers and embryos (Fig. S6), informing relevance of the 3 h time point for this analysis. We therefore applied untargeted metabolomics analysis to mSer, mLiv, eLiv and eCSF from Experiment 2 (Fig. 5*A*) and observed a clear separation between individual sample sets (livers and biofluids) with more subtle changes between treatment and control groups (Figs. 5*B* and S7*A*). Metabolites related to glucocorticoid and kynurenine pathways were observed as major drivers of the separation between all groups and among top significantly changed metabolites (Figs. 5*B* and S7, *A* and *B*). Of note, we detected metabolites from the cortisol group (Fig. S7*C*) and as described above (Figs. S5 and S6) these likely match various sterols. Further work is required to delineate their unique roles in embryonic and maternal tissues. Of note, several sterols followed organ-specific kinetics (Fig. 5, *C* and *D*). A few examples of significant observations include higher embryonic levels of deoxycorticosterol-like while other glucocorticoid pathway metabolites were higher in the maternal organs (Fig. 5*C*), and all detected glucocorticoid pathway metabolites were higher in each of the analyzed organs in the polyI:C-injected compared to saline-injected dams and embryos (Fig. 5*D*). These findings are consistent with reports that glucocorticoid receptors and mineralocorticoid receptors are differentially expressed in tissues across the body and the brain (55). Glucocorticoid-mediated signaling could have important implications for the resolution of the propagation of inflammation in tissues across the maternal-fetal axis.

**Figure 6:**
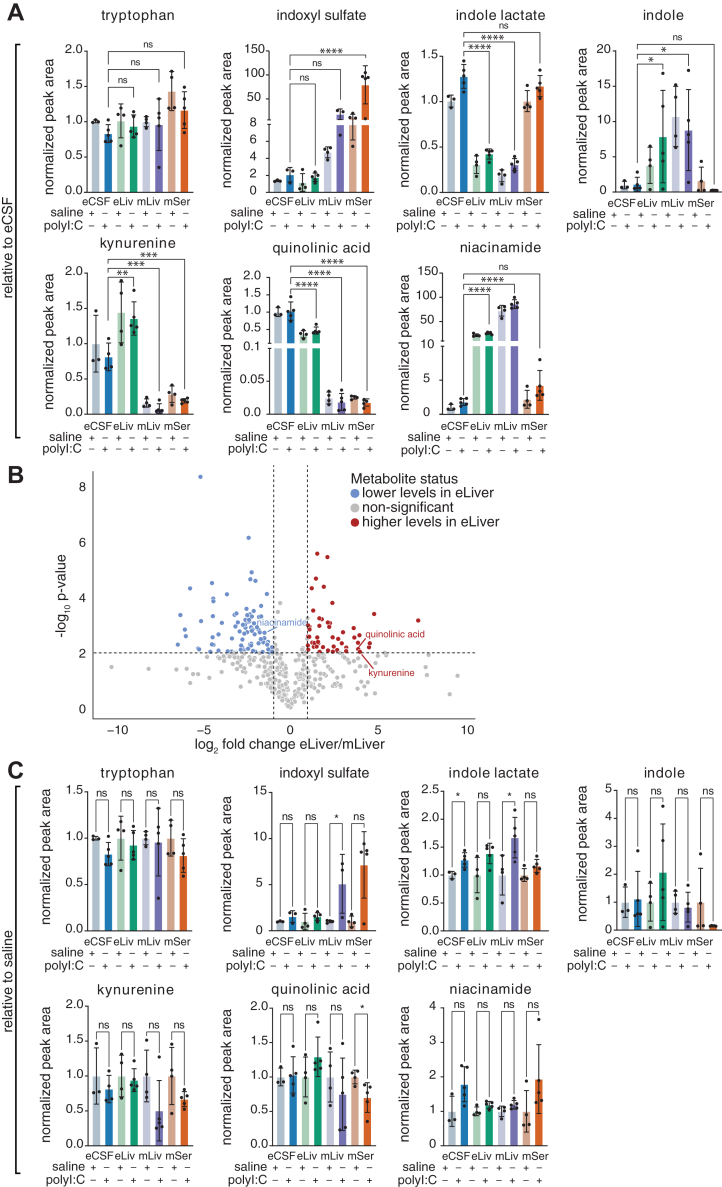
**Metabolic changes in kynurenine pathway metabolites as assessed by untargeted metabolomics in embryonic liver and CSF or maternal liver and serum.***A*, Bar graphs depicting mean and standard deviation levels of indicated metabolites post polyI:C injection for indoxyl and kynurenine pathway intermediates by untargeted metabolomics. ∗ = *p* < 0.01; ∗∗ = *p* < 0.001; ∗∗∗ = *p* < 0.001; ∗∗∗∗ = *p* < 0.0001(one-way ANOVA, Šídák's multiple comparisons test). Graphs are relative to eCSF abundance. *B*, Volcano plot comparing embryonic (eLiver) to maternal (mLiver) liver samples at 3 h post saline injection (each with a minimum of three biological replicates). Untargeted metabolomics at Levels 1 to 3 (exact mass, RT match, MS^2^ match to in-house or online databases and match to eCSF CD-1 library) confidence metabolites were used for this analysis. Metabolites of interest are highlighted (*blue* – lower in eLiver, *red* – higher in eLiver). Only identified kynurenine pathway intermediates are annotated. *C*, Same as A, but graphs are relative to the corresponding control saline sample set.

Our analysis also indicated that each detected metabolite of the kynurenine pathway had an organ-specific distribution (Fig. 6*A*): relative to eCSF indoxyl sulfate was higher in maternal organs; kynurenine and quinolinic acid were higher in the embryonic organs; indole and niacinamide were higher in livers, regardless of embryonic or maternal; indole lactate was lower in the livers (Fig. 6*A*). Broad comparison of embryonic and maternal livers’ metabolome revealed that niacinamide, kynurenine and quinolinic acid were among the top significantly differentially abundant metabolites (Fig. 6*B*), and their metabolic pathway was among the most enriched metabolic pathways in the embryonic liver compared to the maternal liver (Fig. S8). Of note, kynurenine and quinolinic acid were higher in eLiv samples relative to mLiv levels, while niacinamide was higher in mLiv compared to eLiv (Fig. 6, *A* and *B*).

Most kynurenine pathway metabolites were not significantly different in organs between saline and polyI:C injections (Fig. 6*C*). This is aligned with our eCSF data and indicates a later response of this pathway to polyI:C (Fig. 4). Tryptophan levels remained unchanged (Fig. 6, *A* and *C*).

Collectively, our results indicate distinct regulation of the kynurenine pathway in the mother and embryo, with some dysregulation as early as 3 h post-MIA induction, as well as unique persistent differences at 48 h (Fig. 3). Persistent changes could result from maternal inflammation or an embryonic response to maternal stress, since embryonic changes can reflect embryonic metabolism or an overflow of maternal metabolites, and both possibilities likely respond to maternal inflammation.

## Discussion

CSF composition, including proteins and small molecules, is critical for supporting brain development (6), and changes in CSF composition during development may contribute to neuropathology (6, 56). Profiling eCSF composition can reveal abnormalities. For example, we previously identified pro-inflammatory peptides (*e.g.*, cytokines) in eCSF following MIA (35). However, the metabolic profile of the developing eCSF is currently underutilized for uncovering potential effectors of CNS development and disease. There are available mouse models of pathologic conditions that often lead to neurodevelopmental disorders in humans, such as the MIA model, and these can be studied to reveal eCSF metabolomics and the potential role of the metabolic profile of eCSF in neuropathology. However, broad, comprehensive metabolite profiling of mouse eCSF has been extremely challenging in part due to the difficulty in obtaining a sufficient quantity of mouse eCSF. We previously reported polar metabolite profiling comparing adult and embryonic E14.5 CSF, while a study in rats profiled the eCSF proteome on developmental days E17–E20 and focused on the metabolic signature of a rat model of hydrocephalus (57). A recent study employing neuronal imaging in a model of MIA in non-human primates from 6 to 45 months by MRI reported levels of 7 metabolites in the brain including the region of the CSF (58). The study identified acetylaspartate (NAA) as an important metabolite correlating with better cognitive performance and thus a protective mechanism. No metabolite profiling of MIA eCSF has been previously published, even though the metabolite content of the eCSF is likely critical for MIA pathophysiology. The data we report here is from eCSF collection of up to 1 to 2 μL of eCSF from each E14.5 embryo. This sums for about a maximum of 10 to 15 μL of pooled eCSF from a litter of 10 to 12 embryos per mouse. In comparison, we routinely collect 10 to 15 μL CSF from an adult mouse. Nevertheless, our study is first to report deep metabolite profiling of eCSF from two mouse developmental stages, and a comparison of healthy eCSF to eCSF from embryos exposed to MIA. Indeed, our study determined that MIA alters the embryonic mouse CSF metabolome as early as 3 h following maternal injection. Given these limitations of small volume that necessitates litter pooling, our study combined and averaged male and female eCSF. Future studies are needed to examine the causal role sex differences may play in this context.

A limitation of our study stems from the use of polyI:C to model MIA (27, 46, 47). To the best of our abilities, we have controlled for potential biological noise due to inter- and intra-bottle variability by tracking reagent lot number, verifying molecular weight, and tracking endotoxin levels, as recommended in Kowash *et al.*, 2019 (46). Yet, biological noise between and within our experiments was observed and this noise confined the statistical power of our study. In future studies, it will be important to determine the degree to which polyI:C-associated metabolite changes in the eCSF compare with other models of MIA including other forms of maternal illness or environmental teratogens and pollutants (59, 60, 61, 62).

To gain a more complete picture of eCSF metabolite content, we generated a small molecule database of healthy CD-1 eCSF on embryonic day E14.5. This required the development of an experimental pipeline for stringent analysis of eCSF LC-MS data. This database can be used as a refence library of the eCSF metabolite content and is therefore a new resource for researchers in the fields of neurobiology and development. As a proof of concept, we demonstrated the utility of this research tool in a comparison of the eCSF metabolome between two mouse strains (C57Bl/6 and CD-1), two developmental days (E12.5 and E14.5), and in normal vs. pathological conditions (MIA induced by polyI:C exposure).

We found evidence that two developmentally essential metabolic pathways were altered by MIA in the eCSF: the glucocorticoid and kynurenine pathways. First, corticosterone was elevated 3 h following MIA (27, 34, 62), a time prior to the appearance of pro-inflammatory cytokines in the eCSF but coinciding with cytokines and corticosterone elevations in the maternal plasma (62). Glucocorticoids are anti-inflammatory and immunosuppressive and among the most prescribed drugs for the treatment of immune and inflammatory disorders (23). Yet, glucocorticoids (*i.e.*, corticosterone, the main stress hormone utilized in rodents) can be either pro- or anti-inflammatory depending on the tissue type and developmental stage (23, 63, 64, 65). The function of glucocorticoids in the eCSF and potential role(s) of these small molecules in shaping the neurocognitive effects of maternal inflammation are not known.

We speculate that maternal serum could contribute to the corticosterone levels in eCSF, since stress in mothers can downregulate placental 11β-hydroxysteroid dehydrogenase 2 (11β-HSD2), an enzyme that converts corticosterone into its inert form (64, 66). Thus, MIA could provide the conditions necessary for maternally derived corticosterone to cross the placenta into fetal circulation and enter the eCSF (64). Once in the brain, corticosterone is a potential candidate for mediating the effects of MIA on embryonic brain development by multiple mechanisms, such as accelerating neural progenitor proliferation (67, 68). Glucocorticoids signal through glucocorticoid receptor (GR) and mineralocorticoid receptor (MR), both of which are differentially expressed in tissues across the body and the brain (55, 69, 70) and are reported to have opposing roles during inflammation and stress (55, 70). Thus, future studies will be necessary to determine if eCSF-corticosterone directly impacts forebrain neurogenesis in the context of MIA.

A second set of metabolites that we found to be affected in eCSF following MIA originate from tryptophan catabolism and include kynurenine, kynurenic acid, and quinolinic acid (47, 49). The kynurenine pathway was altered 48 h following MIA, a time that coincides with elevated cytokine levels in eCSF (35) and brain (34, 27), which are indicative of embryonic inflammation. Kynurenine is a downstream metabolite in the catabolism of the essential amino acid tryptophan. Tryptophan is a precursor to serotonin (5-HT), a neurotransmitter with key roles in regulating neurodevelopmental processes including circuit formation (47, 50, 71). However, depending on the tissue or context, it is estimated that up to 90% of tryptophan is catabolized to kynurenine (72), a well-documented anti-inflammatory metabolite with direct effects on both brain and immune function (73, 74, 75). Other metabolites in the kynurenine metabolism pathway, *e.g.* kynurenic acid and quinolinic acid, are also immunomodulatory (75). Notably, altered kynurenine pathway metabolism in adult patients has been associated with neurodevelopmental disorders such as schizophrenia (76, 77, 78, 79) autism spectrum (25), and bipolar disorder with a history of psychosis (80). Further, kynurenine-related metabolites were shown to be useful biomarkers for viral and bacterial CNS infections (81, 82).

Whether the source of eCSF kynurenine is maternal or embryonic is currently unclear. Kynurenine can pass from maternal plasma (49) to be metabolized by the placenta (47) or could be metabolized more locally by the maturing embryonic liver (83). eCSF kynurenine can also be metabolized locally in the embryonic CNS (75). Once in the eCSF, changes in metabolites of the kynurenine pathway have the potential to mediate MIA effects on neurodevelopment. For example, kynurenic acid and quinolinic acid are both ligands for the N-methyl d-aspartate receptor (NMDAR), which modulates the proliferation and maturation of glutamatergic cells (84). Yet, kynurenic acid and quinolinic acid exert opposing effects upon NMDAR, and the balance of these two metabolites is crucial for normal glutamatergic neurotransmission (85) whereas their imbalance is implicated in neurodevelopmental disorders (86). Furthermore, it has been shown that glutamatergic cells are affected in MIA-induced cerebral cortical disorganization models (33, 34, 35). This suggests a potential link between metabolites of the kynurenine pathway and disrupted cerebral cortical development.

Therefore, our eCSF metabolite reference library is a resource that can be used to study MIA pathology. In our examples, we show directions of investigation that include routes by which peripheral metabolites gain access to the eCSF and embryonic brain (1, 87, 88, 89, 90, 91), the kinetics of a developing embryo sensing and acquiring maternal illness, and how long does the impact of a maternal infection persist in the developing embryo, despite maternal recovery. Indeed, our data suggest that the dynamics of maternal-fetal illness differ by different metabolic pathways. The corticosterone pathway provides an early indication of maternal illness in the developing embryo whereas other metrics of inflammation, including kynurenine, and previously published cytokine profiles (35), contribute to later inflammatory signatures of eCSF. Notably, many metabolites detected at E12.5 were significantly less abundant at E14.5 (Fig. 2*D*). Future analyses will determine if the apparently reduced abundance was due to further metabolism of pathway intermediates, a dilution effect due to increased embryo growth/CSF volume, or some level of CSF turnover already present at this early age.

Importantly, the polyI:C MIA model represents just one available model for investigating the consequences of maternal illness on the developing brain. The metabolic maternal-fetal axis is sensitive to perturbation from many types of challenges including other forms of maternal illness, diet, substance abuse, and environmental teratogens, all of which are associated with manifestations of later-onset neurologic and psychiatric conditions. Thus, our resource takes a first step towards elucidating how the eCSF is altered in MIA and provides a path forward for investigating whether similar mechanisms and kinetics generalize beyond MIA to other conditions as well.

## Experimental procedures

### Institutional review Board Statement

This research complies with all relevant ethical regulations and was approved by Boston Children’s Hospital IACUC and Boston Children’s Hospital Institutional Biosafety Committee. All experiments involving mice in this study were approved by Boston Children’s Hospital IACUC.

### Animals

All mouse work was performed in accordance with the Institutional Animal Care and Use Committees (IACUC) and relevant guidelines of Boston Children’s Hospital. Embryonic day (E) 14.5 embryos from time-pregnant CD-1 (ICR) dams were purchased from Charles River Laboratories (CRL) for the baseline conditions metabolomics database and maternal kynurenine injection time-course experiments. E12.5 embryos from timed-pregnant C57BL/6 dams were purchased from CRL for maternal immune activation metabolomics experiments. For timed pregnancies, females were checked for the presence of plugs, and the date of the plug was noted as embryonic day 0.5 (E0.5). All animals were housed under 12 h/12 h day–night cycle with access to standard chow and water ad libitum.

### Embryonic and maternal body fluid and tissue collection and analysis

Embryonic CSF (eCSF) was collected by inserting a glass capillary into the cisterna magna and processed as described in the text as well as stepwise procedural video (92). CSF samples were pooled across litters (single litter per pool), and immediately frozen on dry ice. Pooling resulted in a maximum of 10 to 15 μl eCSF from each litter. Embryonic blood was collected by glass capillary following a neck artery nick and pooled per litters. Maternal blood was collected by tail vein nick for non-terminal time points and cardiac puncture for terminal time points.

### Maternal immune activation

On E12.5, pregnant dams received a single dose (20 mg/kg, i.p.) of polyinosinic-polycytidylic acid (polyI:C, Sigma Aldrich) or sterile saline as the vehicle control. To confirm maternal immune activation, maternal blood was collected at 3 h following maternal polyI:C injection by either tail-nick or intracardiac puncture. Maternal blood was also collected 48 h following maternal polyI:C injection by terminal intracardiac puncture. After maternal blood samples were coagulated and centrifuged, 3 h and 48 h MIA serum samples were analyzed by LEGENDplex Mouse Inflammation Panel (13-plex with V-bottom Plate) FACS-ELISA (BioLegend). Validation of MIA was performed by measurements of cytokine levels in maternal serum using FACS-ELISA. Results reported in Fig. S3*B* are from mice that are the source of the data of experiment 1 harvested at 48 h that is reported in Figure 3, *G* and *I*. Results reported in Fig. S3*D* are from mice that are the source of the data of experiment 2 harvested at 48 h that is reported in Figure 3, *H* and *J*. Statistical analyses were performed using unpaired t-tests through the GraphPad Prism 9.2.0 and Prism 10 software.

### Sample preparation for LC-MS analysis of polar metabolites from CSF, plasma, and liver from embryonic and adult mice

We used fresh frozen tissues or processed (see method above) CSF and plasma. Per condition, 2 to 8 μl embryonic CSF, 5 μl plasma, approximately 0.5 × 0.2 cm sized sample of maternal liver, and combined embryonic livers (each the size of about 1 × 1 mm) were extracted. For eCSF, per experiment volumes were kept consistent across samples and whenever possible adjusted to the lowest amount available. Samples were processed for metabolomics with similar rigor as described (93). Tissues were homogenized with a handheld homogenizer (Sigma, Z359971). 300 μl of extraction solvent (80% LC/MS-grade methanol, 20% 25 mM Ammonium Acetate, and 2.5 mM Na-Ascorbate prepared in LC/MS water and supplemented with isotopically labeled internal standards (17 amino acids and isotopically labeled reduced glutathione, Cambridge Isotope Laboratories, MSK-A2-1.2 and CNLM-6245-10)) was used per sample. After centrifugation for 10 min at maximum speed on a benchtop centrifuge (Eppendorf) the cleared supernatant was dried using a nitrogen dryer (ThermoFisher Scientific, TS-18826) and reconstituted in 17 μl (eCSF) or 25ul (plasma, tissues) “QReSS” water (supplemented with QReSS, Cambridge Isotope Laboratories, MSK-QRESS-KIT) by brief vortexing. Extracted metabolites were spun again and the cleared supernatant was transferred to LC-MS microvials. A small amount of each sample was pooled and serially diluted 3 and 10-fold for quality controls (pool-QC) throughout the run of each batch. About half of eCSF (7 μl), 1/25th of plasma (1 μl), and 1/25th of tissue (1 μl) were injected into a chromatographic column and subsequently analyzed using LCMS.

### Chromatographic conditions for LC-MS

ZIC-pHILIC chromatography was used for polar metabolites: an appropriate volume of reconstituted sample was injected into a ZIC-pHILIC 150 × 2.1 mm (5 μm particle size) column (EMD Millipore) operated on a Vanquish Flex UHPLC Systems (Thermo Fisher Scientific, San Jose, CA). Samples were analyzed with similar rigor as described (93). Chromatographic separation was achieved under the following conditions: buffer A was acetonitrile; buffer B was 20 mM ammonium carbonate, 0.1% ammonium hydroxide. Gradient conditions were: linear gradient from 20% to 80% B; 20 to 20.5 min: from 80% to 20% B; 20.5 to 28 min: hold at 20% B. The column oven and autosampler tray were held at 25 °C and 4 °C, respectively. Chromatographic performance was quality controlled using a mixture of unlabeled standard amino acids and a mixture of chemically diverse compounds (Cambridge Isotope Laboratories, MSK-A2-US-1.2, and MSK-QRESS-US-KIT) with 1 μl of each injected after every run on our HILIC method.

### MS data acquisition conditions for targeted or untargeted analysis of polar metabolites

MS data acquisition was performed using a QExactive benchtop orbitrap mass spectrometer equipped with an Ion Max source and a HESI II probe (Thermo Fisher Scientific) and was performed in positive and negative ionization mode in a range of m/z = 70 to 1000, with the resolution set at 70,000, the AGC target at 1 x 10^6^, and the maximum injection time (Max IT) at 20 msec. Optimal HESI conditions were determined utilizing a mix of chemical standards: Sheath gas flow rate: 35; Aug gas flow rate: 8; Sweet gas flow rate: 1; Spray voltage: 3.5 kV (pos), 2.8 kV (neg); Capillary temperature: 320 °C; S-lens RF: 50; Aux gas heater temperature: 350 °C. For untargeted analysis, an additional Top8 method (see below) was employed in either positive or negative mode with ddMS settings as: the resolution set at 17,500, the AGC target at 1 × 10^5^, the maximum IT at 50 msec, the isolation window at 1.0 m/z, and stepped NCE at 20,40, and 80.

As our software/hardware did not allow for the widely employed AquireX assisted MS^2^ inclusion/exclusion list data collection, we performed repeated data collections with the following logic: At the end of each experimental batch spectral data was collected in both positive and negative mode on additional injections of the pool-QC. We collected spectral data in a data-dependent acquisition mode (DDA) such that for every MS^1^-level scan the top 8 most abundant ions were picked for fragmentation (Top8-level experiment), an exclusion window was set to 6 s, such that ions picked for fragmentation would be put on an exclusion list for 6 s before they could be picked for fragmentation again. This allowed for the collection of about 300 to 500 individual ion spectral scans per sample injection. Then, MS^1^ and MS^2^ level data were quickly analyzed using CompoundDiscoverer and detected features were used to create an inclusion and exclusion list (per polarity) that ensured maximal inclusion of interesting features (features with signals above mock level and with no MS^2^ spectra collected) and maximal exclusion of non-interesting features (features present in mock samples for which MS^2^ spectra was already collected). This strategy was then performed iteratively up to 4 times. We noted an incremental increase in the number of features with associated spectra generated with this strategy; however, we note that this strategy did not perform as well as reported from an AquireX type of experiment.

### Generation of targeted metabolomics libraries (in-house MS^1^ and MS^2^-level databases)

Two separate MS^1^ and MS^2^-level libraries were generated. A set of standards premixed in 7 individual pools were run on our LC-MS HILIC method. Information on these pool sets has been previously published (93, 94). Briefly, we first collected MS^1^ level information (retention time and peak shape and quality were noted, see Table S1 see also Supporting Dataset S3). To generate the MS^2^-level database, each pool set was injected twice (for positive and negative mode), and specific inclusion lists were preset for a PRM-type experiment. This inclusion list was based on the already determined retention times and was focused on compounds with peaks of minimum acceptable quality. Thus, high-quality MS^2^ data were collected per individual metabolite and MS^2^ spectra were averaged out from three different HCD cell energies: 20, 40, 80 NCE. We named this database “HILIC_all” (Supporting Dataset S1). The second library set was based on the MSMLS library of compounds (MSMLS, IROA Technologies’), which we named “MSMLS_HILIC”. Compounds were solubilized and pooled based on the manufacturer’s recommendations. Each pool was run as an individual injection and MS^1^ information (retention time and peak shape and quality) was noted. (Table S2 and see also Supporting Dataset S4). Then, spectral MS^2^ information was collected at both ionization modes as above with the exception that individual spectra were not averaged but collected at the three different HDC energies independently (Supporting Dataset S2). All spectra were manually inspected and compared to known and well-characterized spectral databases (METLIN, MONA, and mzCloud). Most of the spectra included in our library were thus verified. Where no match was found but the spectrum was of high quality, we included it in the library and added a note in our metadata file (Supporting Dataset S3 (HILIC_all) and Supporting Dataset S4 (MSMLS_HILIC)). Few metabolites were also run from individual wells, to try and obtain better signal-to-noise ratios. Overall, our database included: HILIC_all MS^1^ – 261 compounds, MS^2^ – 144 compounds; MSMLS_HILIC MS^1^ – 236 compounds; MS^2^ – 307 compounds. The overlap between the two MS^1^ databases was about 100 compounds.

### Targeted metabolomics data analysis

Relative quantification of polar metabolites was performed with TraceFinder 5.1 (Thermo Fisher Scientific, Waltham, MA, USA) using a 5 ppm mass tolerance and referencing an in-house library of chemical standards (HILIC_all and MSMLS_HLIC). We routinely queried about 257 compounds (37 internal standards and 220 metabolites). Pooled samples and fractional dilutions were prepared as quality controls and injected at the beginning and end of each run. In addition, pooled samples were interspersed throughout the run to control for technical drift in signal quality as well as to serve to assess the coefficient of variability (CV) for each metabolite. Data from TraceFinder were further consolidated and normalized with an in-house R script: (https://github.com/FrozenGas/KanarekLabTraceFinderRScripts/blob/main/MS_data_script_v2.4_20221018.R). Briefly, this script performs normalization and quality control steps: 1) extracts and combines the peak areas from TraceFinder output.csvs; 2) calculates and normalizes to an averaged factor from all mean-centered chromatographic peak areas of isotopically labeled amino acid and QReSS internal standards within each sample; 3) filters out low-quality metabolites based on user-entered cut-offs calculated from pool reinjections and pool dilutions; 4) calculates and normalizes for biological material amounts based on the total integrated peak area values of high-confidence metabolites. In this study, the linear correlation between the dilution factor and the peak area cut-offs are set to RSQ>0.95 and the coefficient of variation (CV) < 30%. Finally, data were log-transformed and Pareto scaled within the MetaboAnalyst-based statistical analysis platform (95) to generate PCA, PLSDA, volcano plots, and heatmaps. Individual metabolite bar plots and statistics were calculated in Excel and GraphPad Prism. Finally, for statistical analysis within MetaboAnalyst missing values were filled using LoDs (one-fifth of the minimum positive value of each variable) while when plotting individual metabolites (using GraphPad Prism) missing values were omitted from analysis.

### Untargeted metabolomics data analysis using CompoundDiscoverer (mock-filtering mode)

Relative quantification for untargeted polar metabolomics was performed with Compound Discoverer (CD) 3.3. A general workflow was built to best suit our polar metabolomics LC-MS method (details on each parameter are given in Supporting Dataset S5). Positive and negative modes were analyzed separately. Both MS^1^ and MS^2^-level in-house databases were used (HILIC_all and MSMLS_HLIC). For the analysis of MIA samples, additionally, MS^1^ and MS^2^-level CD-1 DBs were used (in positive and negative modes, see above). Filtering steps were performed based on peak noise levels, ppm error, formula annotation, and the relative abundance of the integrated peak area in true samples compared to mock samples, where features >3-fold higher in samples were retained. Normalization steps were performed based on a “scaling factor”: a per-sample normalizer. This scaling factor was determined per experiment or sample comparison: each sample was analyzed by targeted metabolomics first, and the scaling factor corresponded to the biological material normalizer from the total integrated peak area of mean-centered values from high-confidence metabolites. This normalizer rarely differed more than 2-fold within a sample type. When experiments from different experimental days were compared (such as CD-1 to C57BL/6 comparison, as in Fig. 2), untargeted analysis was performed on the combined samples, and data were independently curated. This ensured adequate chromatographic retention time correction and alignment and thus proper feature alignment, identification, and annotation. Within our HILIC chromatography, we rarely observe chromatographic shifts larger that 1 min and retention times drifts larger than 40 s. We further observed that a larger than 40 s threshold for retention time shift led to mis-annotation for our in-house library compound databases (for example betaine vs valine or alanine vs beta-alanine). Therefore, these parameters were set as limits for retention time correction and matching to internal databases. Post CompoundDiscoverer analysis included merging of negative and positive mode data and statistical analysis using the MetaboAnalyst platform. As compound annotation at levels higher than Level 1 includes a significant level of uncertainty, we decided not to merge features based on annotation name but rather to retain information from both positive and negative mode analyzed data (thus multiple compounds are annotated as either “Metabolite_name_[M-H]” or “Metabolite_name” for negative or positive mode respectively, for example, Fig. 2*D*). Finally, data were log-transformed and Pareto scaled within the MetaboAnalyst-based statistical analysis platform (95) to generate PCA, PLSDA, volcano plots, and heatmaps. Individual metabolite bar plots and statistics were calculated in Excel and GraphPad Prism. Pathway enrichment was similarly performed within MetaboAnalyst following the above data transformation steps.

### Untargeted metabolomics data analysis using ClusterFinder and IROA credentialing (IROA mode)

All steps for sample preparation for IROA-assisted metabolomics were as described above except for the final resuspension step, which was performed using the IROA provided Internal Standard (IS) (95% ^13^C-labeled, IROA TruQuant Yeast Extract Semi-targeted QC Workflow, IROA Technologies). Relative quantification for untargeted polar metabolomics was performed with CD 3.3 (for mock-filtering mode) and ClusterFinder 4.2.23 (for IROA-mode). We initially determined that 7ul of CSF reconstituted in 20 μl IS led to optimal signal intensity correspondence between labeled and unlabeled isotopologues (Fig. S1*B*, step 1). Default parameters were set for each search (targeted or untargeted) except for the minimal signal intensity threshold, which was set lower, at 10.000. Additionally, we integrated our targeted metabolomics MS^1^-level databases into ClusterFinder and thus performed a secondary level of annotation within the software. Retention time correction and peak integration were performed within the software and were not further curated.

### Generation of untargeted metabolomics CD-1 library (CD-1 DB MS^1^ and MS^2^-level database)

Embryonic CSF from E14.5 embryos was collected as described above. 7 mice were used and CSF from all embryos was pooled per mouse. For each replicate, 3 μl was used for IROA-assisted analysis and 3 μl for mock-filtering analysis. As IROA analysis did not yield satisfactory annotation we only generated eCSF-specific metabolite databases from mock-filtering strategy. Within CD, we followed the steps outlined above. We extensively curated each feature. Features were given identifying tags that would help organize certainty of identification and annotation (Level 1-4). All features with matches to our in-house database were confirmed after manual inspection and any double annotations were removed (as well as renamed and specifically tagged) if retention times were not ideal match. Both MS^1^ and MS^2^ level databases were generated within CD on curated data. After export of MS^2^ library to mzVault, the CD-1 eCSF database was further manually curated, where low-quality spectra were removed (low signal to noise). Of note, most features are represented by a single collected spectrum.

## Data availability

All data generated as part of this study is deposited at Metabolomics Workbench https://doi.org/10.21228/M8ST6K, project ID: PR001918.

## Supporting information

This article contains supporting information.

## Conflict of interest

The authors declare the following financial interests/personal relationships which may be considered as potential competing interests: J.C. has been an employee of Dyne Therapeutics since April 2021. Other authors declare no conflict of interest.
