# Supplementary material for: Profiling metabolome of mouse embryonic cerebrospinal fluid following maternal immune activation
Source: J Biol Chem. 2024 Sep 7;300(10):107749. doi: 10.1016/j.jbc.2024.107749 (PMC11497393; doi:10.1016/j.jbc.2024.107749)
Supplement: Supplementary Information [file mmc1.pdf]

Supplementary Information for:

Profiling metabolome of mouse embryonic cerebrospinal fluid following maternal immune activation

Boryana Petrova[1,2]*†, Tiara E Lacey[1,3]*, Andrew J Culhane[1], Jin Cui[1], Jeannette R Brook[1], Alexander Raskind[4], Aditya Misra[5], Maria K Lehtinen[1,2] and Naama Kanarek[1,2,6]††

* - co-first
† - co-corresponding

Author Affiliations
1. Department of Pathology, Boston Children's Hospital, Boston, MA 02115, USA;
2. Harvard Medical School, Boston, MA, USA 02115;
3. Graduate Program in Biological and Biomedical Sciences, Harvard Medical School, Boston, MA 02115, USA; Boston, MA 02115;
4. IROA Technologies™ LLC, Chapel Hill, NC, USA
5. Harvard-MIT Division of Health Sciences and Technology; Massachusetts Institute of Technology, Cambridge, MA 02139
6. Broad Institute of Harvard and Massachusetts Institute of Technology, Cambridge, MA 02142, USA

† Corresponding author. boryana.petrova@childrens.harvard.edu; +1-617-355-7433
†† Corresponding author. naama.kanarek@childrens.harvard.edu; +1-617-919-7352

Figure S1

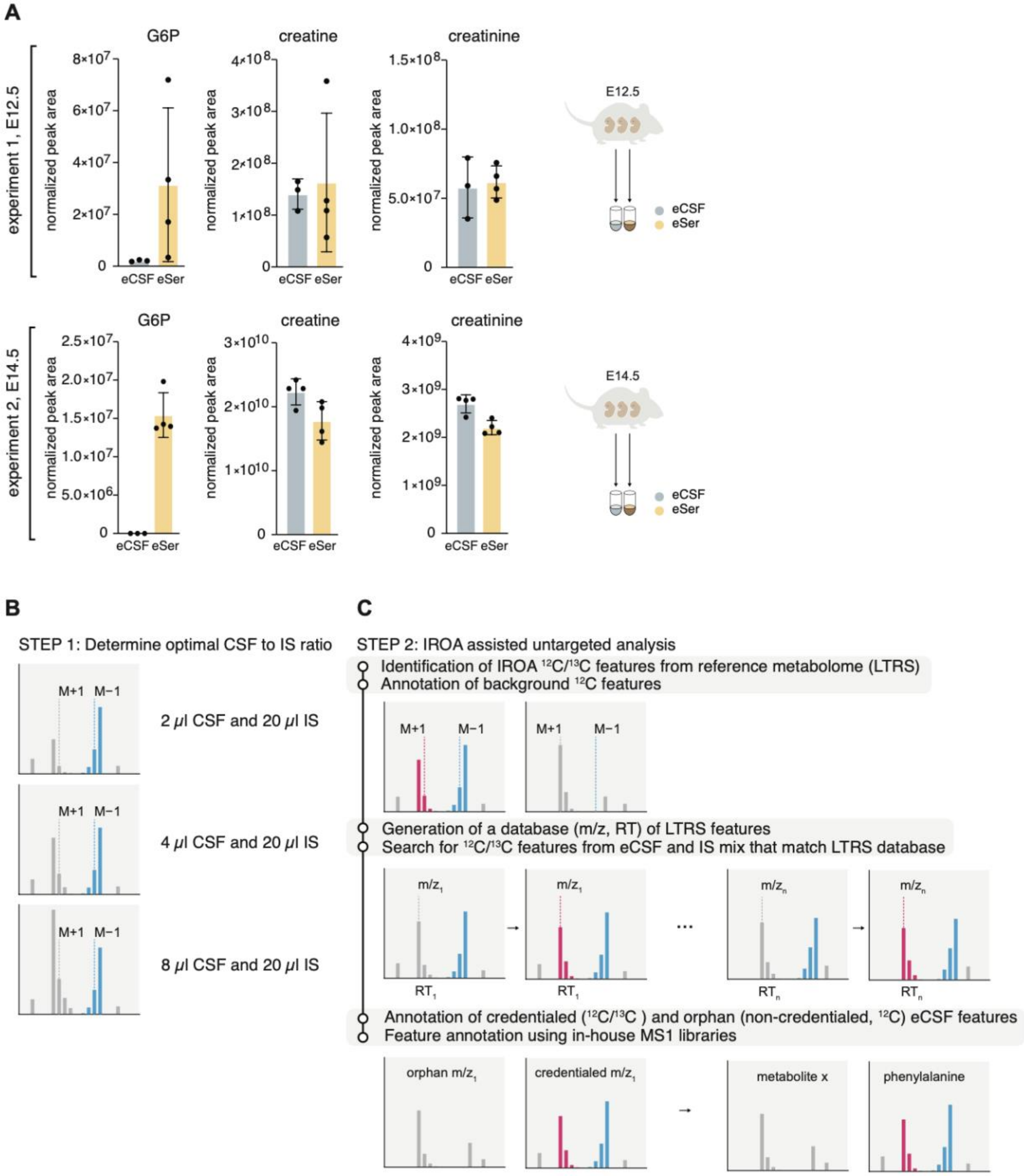

Figure S1. Overview of IROA^{R}-assisted untargeted analysis experimental steps.

A. Validation of embryonic CSF (eCSF) purity by assessment of CSF metabolites and cellular metabolites precent in red blood cells that report blood contamination of CSF samples. Presented is the comparison between eCSF and embryonic serum (eSer) for the two indicated embryonic stages. Glucose-6-phosphate (G6P) is a glycolysis metabolite not present in the acellular CSF, but is present from leakage of red blood cells in the serum. Creatine and creatinine are predicted

to be present in both the CSF and serum. Bar graphs depicting mean +/- standard deviation for indicated metabolites. eCSF and eSer were normalized to account for volume differences to allow for relative comparisons. For experiment 2, at E14.5, G6P was not detected at all, and the graph depicts zero values. Embryonic CSF and serum were pooled per litter across each analysis. Each measurement represents an independent mouse or litter.

B. Schematic representation of step one of IROA analysis, where different amounts of mouse adult CSF (depicted in grey) were mixed with fixed amount of IROA internal standards (IS, at 5:95 unlabeled (^{12}C) and ^{13}C-labeled metabolome, as depicted in blue). This determined the optimal ratio for our work (4 µl CSF to 20 µl IS) – where majority of detected features were observed at one-to-one ratio.

C. Schematic representation of the second step of IROA analysis (that yielded the data presented in Fig 1D): a database of compounds based on untargeted LC-MS metabolomics was generated using a standard created by IROA^R: long-term reference sample (LTRS), that is a fully labeled *S. cerevisiae* yeast cell extract, used as a 1/1 mix of 95:5/5:95 unlabeled (^{12}C) and ^{13}C-labeled reference metabolome (^{12}C/^{13}C reference). Only features with credentialed signal, that is comprised of labeled and unlabeled isotopologues, were considered. Then embryonic CSF (eCSF) samples prepared using the optimal ratio of IS to CSF were queried in a quasi-targeted manner – referencing the LTRS database. Features were further annotated using in-house databases. Phenylalanine is given as an example of credentialed feature, metabolite x – as an example of non-credentialed (orphan m/z) signal.

Figure S2

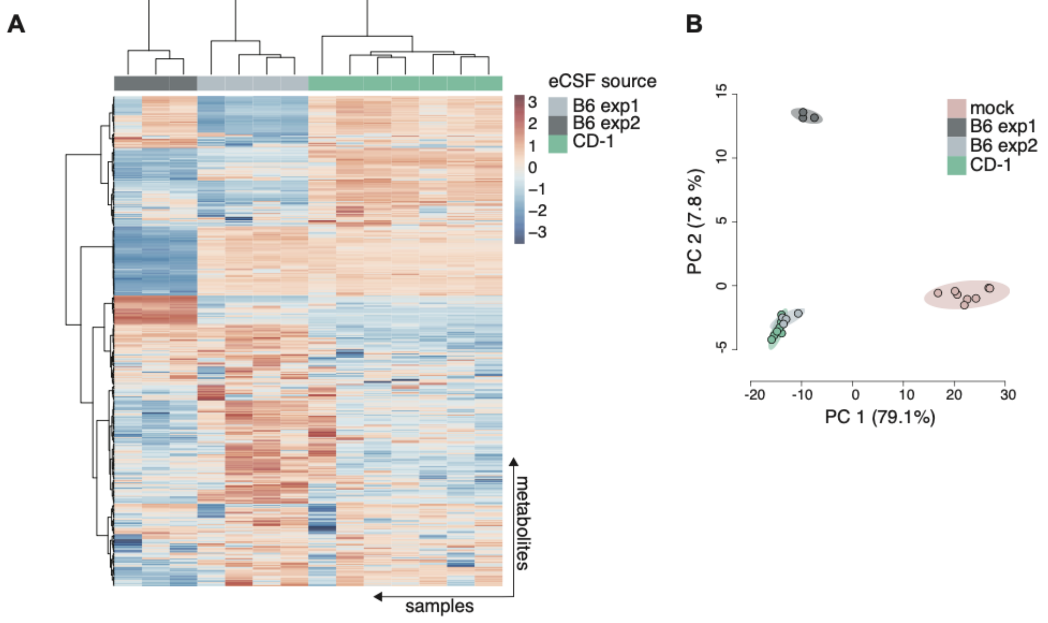

Figure S2. Untargeted metabolomics of eCSF from two mouse strains, CD-1 and C57Bl/6. A, B Heatmap (A) and PCA (B) plots of untargeted metabolomics data from CD-1 and C57Bl/6 eCSF at embryonic day E14.5 (with a minimum of four biological replicates per condition, mock samples were prepared in a technical triplicate). Plots were generated using the online MetaboAnalyst tool, after log transformation and Pareto scaling of the combined data from normalized positive and negative-mode analysis. Compounds present in the in-house and CD-1-based eCSF libraries (at MS^1-level) were used for the heatmap comparison (A) while level 1-3 confidence metabolites (including matches to the CD-1 database presented in Figure 1) were used for the PCA in B. In B, also mock samples were included for comparison.

Figure S3

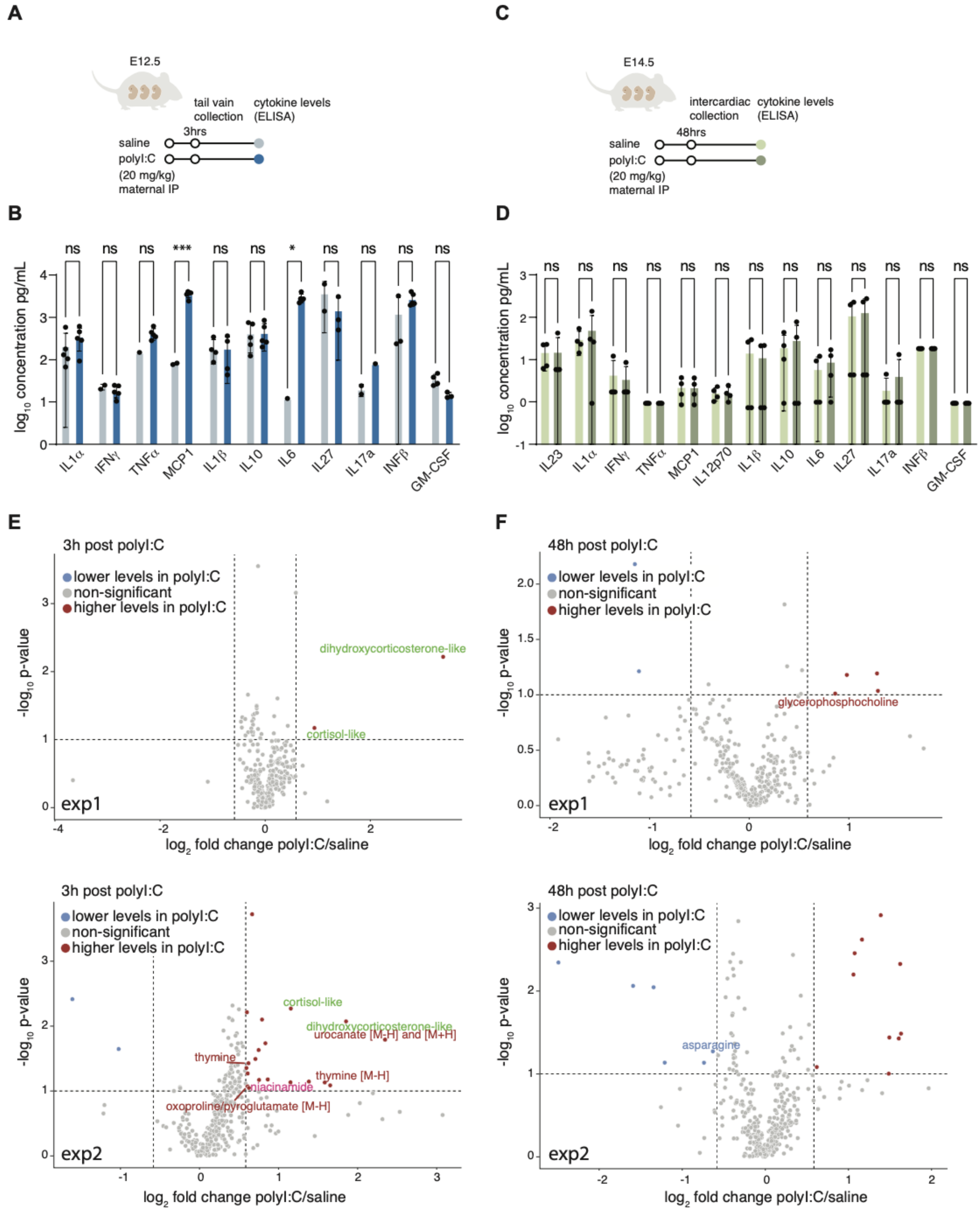

Figure S3. Application of eCSF metabolome reference library in the study of MIA mouse model.

A Schematic of the experimental workflow for testing the inflammatory response in the maternal serum 3hrs post polyI:C injection.

B Assessment of cytokine levels in maternal serum 3hrs post polyI:C delivery using ELISA. Graphs represent mean +/- standard deviation. *= $p<0.01$; **= $p<0.001$; ***= $p<0.001$; n.s.=not significant, by unpaired t test for each cytokine.

C, D Same as A and B, but for assessment of inflammatory response 48hrs post polyI:C delivery. Graphs represent mean +/- standard deviation. n.s.= not significant by unpaired t test for each cytokine.

E, F Volcano plots of untargeted metabolomics analysis of eCSF metabolites plotted as abundance of metabolites in the polyI:C- over saline-injected samples. Data from experiments 1 and 2 are presented separately, at two time points, 3hrs (E,) and 48hrs (F) post injection (a minimum of three biological replicates per condition, per experiment). Significantly changed metabolites are highlighted, only metabolites present in our in-house database are annotated. Blue- lower in polyI:C-injected cohort, red – higher in polyI:C-injected cohort. Some metabolites of interest are in green (glucocorticoid pathway) and magenta (kynurenine pathway). Data were analyzed using the online MetaboAnalyst tool, after log transformation and Pareto scaling of normalized positive and negative-mode combined datasets. Metabolites from Levels 1-3 (including matches to the CD-1 database presented in Figure 1) confidence were used for this analysis.

Figure S4

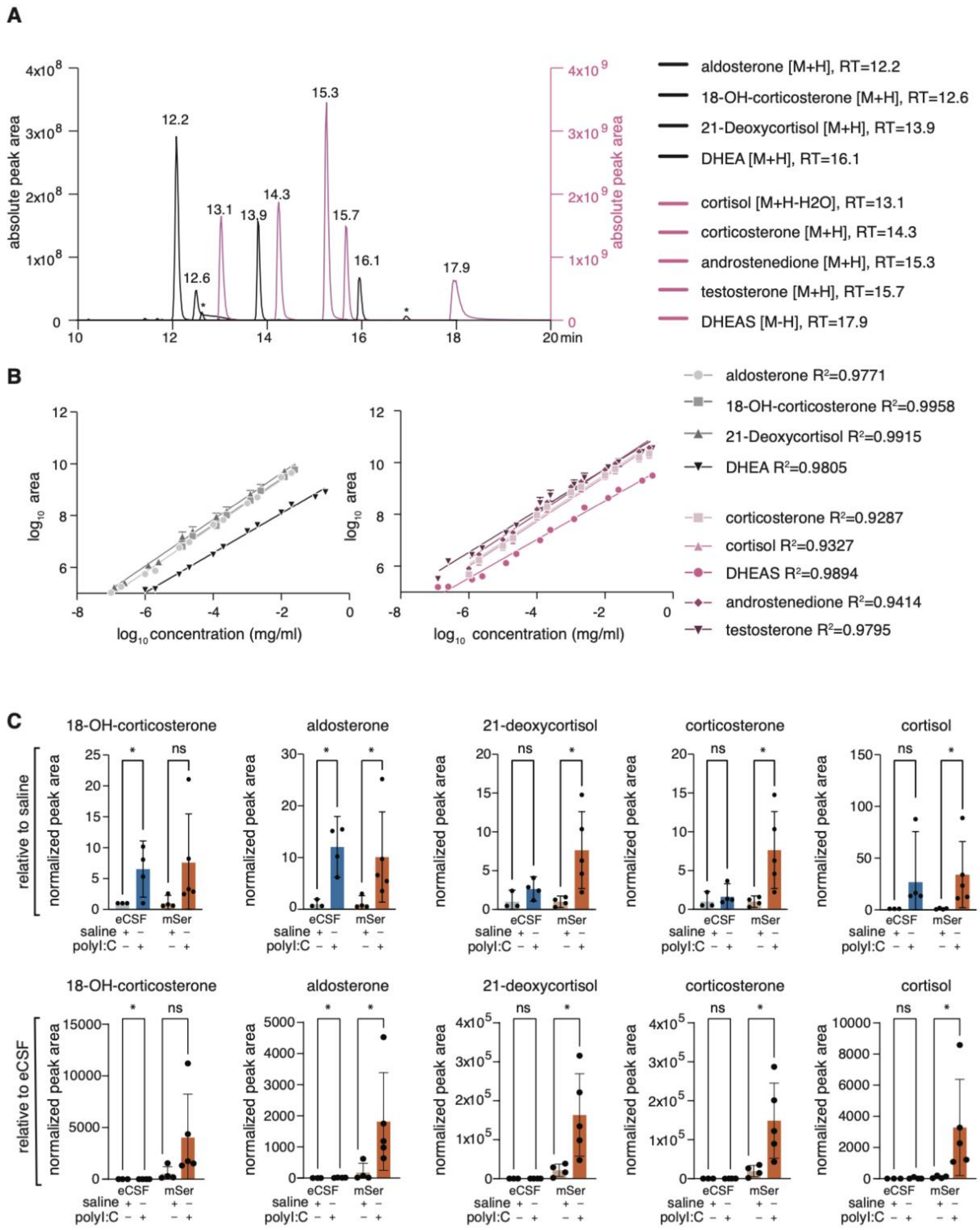

Figure S4. A targeted metabolomics method for detection of glucocorticoids by LC-MS.

A Retention times of indicated glucocorticoid-related metabolites detected using standards are shown. Peaks are grouped based on absolute signal intensity: low (black) and high (magenta). Individual retention times as well as preferred adducts are indicated. Two low-intensity peaks indicated with a star are unknown impurities associated with the aldosterone m/z. “OH” denotes hydroxy

B Linear dynamic range for individual glucocorticoid-related metabolites measured for their standards. R^2 values are indicated. Groups are as in A. Presented are the average values (from two independent dilution series) and standard deviation of logged intensity (absolute counts) and concertation (in mg/ml). “OH” denotes hydroxy

C Glucocorticoid-related metabolites post polyI:C injection from eCSF or maternal serum (mSer). Metabolites levels are depicted as mean and standard deviation. *= $p<0.05$ by unpaired t-test. Graphs are presented relative to corresponding control, saline-injected samples. “OH” denotes hydroxy

Figure S5

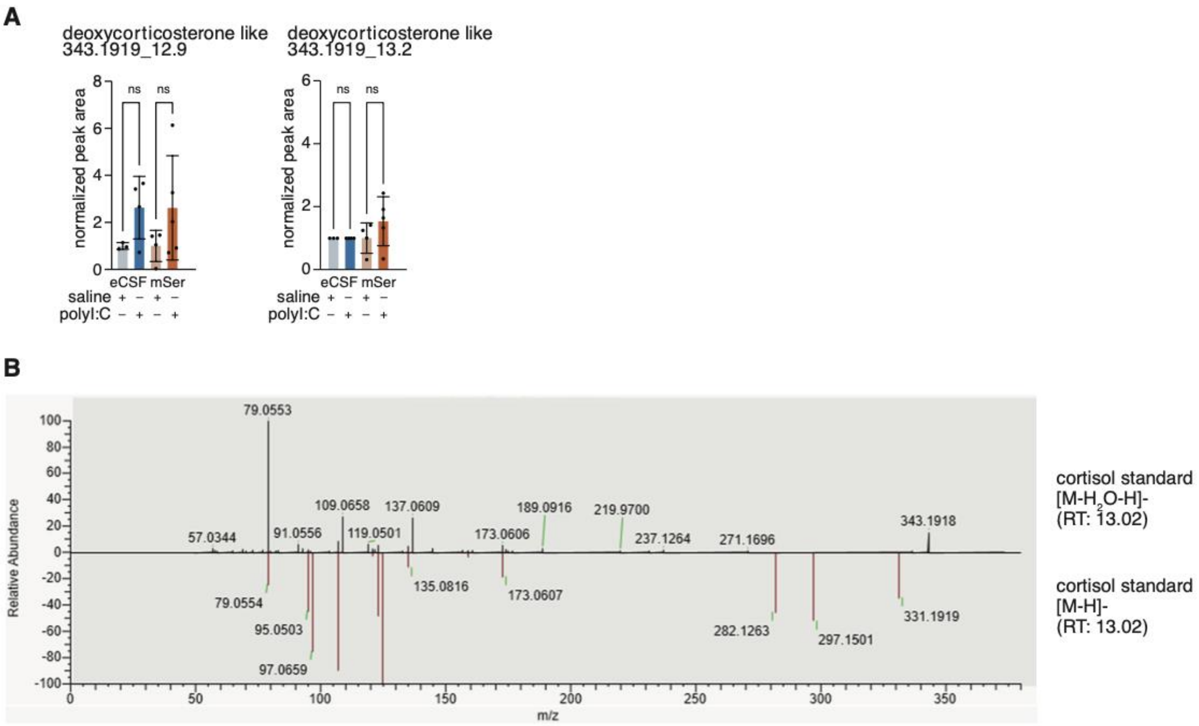

Figure S5. Chemical annotation of two metabolites detected by untargeted analysis of eCSF.

A The indicated LC-MS features as detected post polyI:C injection from eCSF or maternal serum (mSer). The features are at m/z = 343.1919 at 12.9 and 13.2 min retention time as detected using our glucocorticoid method on negative ionization mode (Supplementary Fig. S4). Bar graphs depict mean and standard deviation levels of the features. ns = non-significant by unpaired t-test.

B MS^2 spectral information of two cortisol adducts detected with our LC-MS method from a chemical standard.

Figure S6

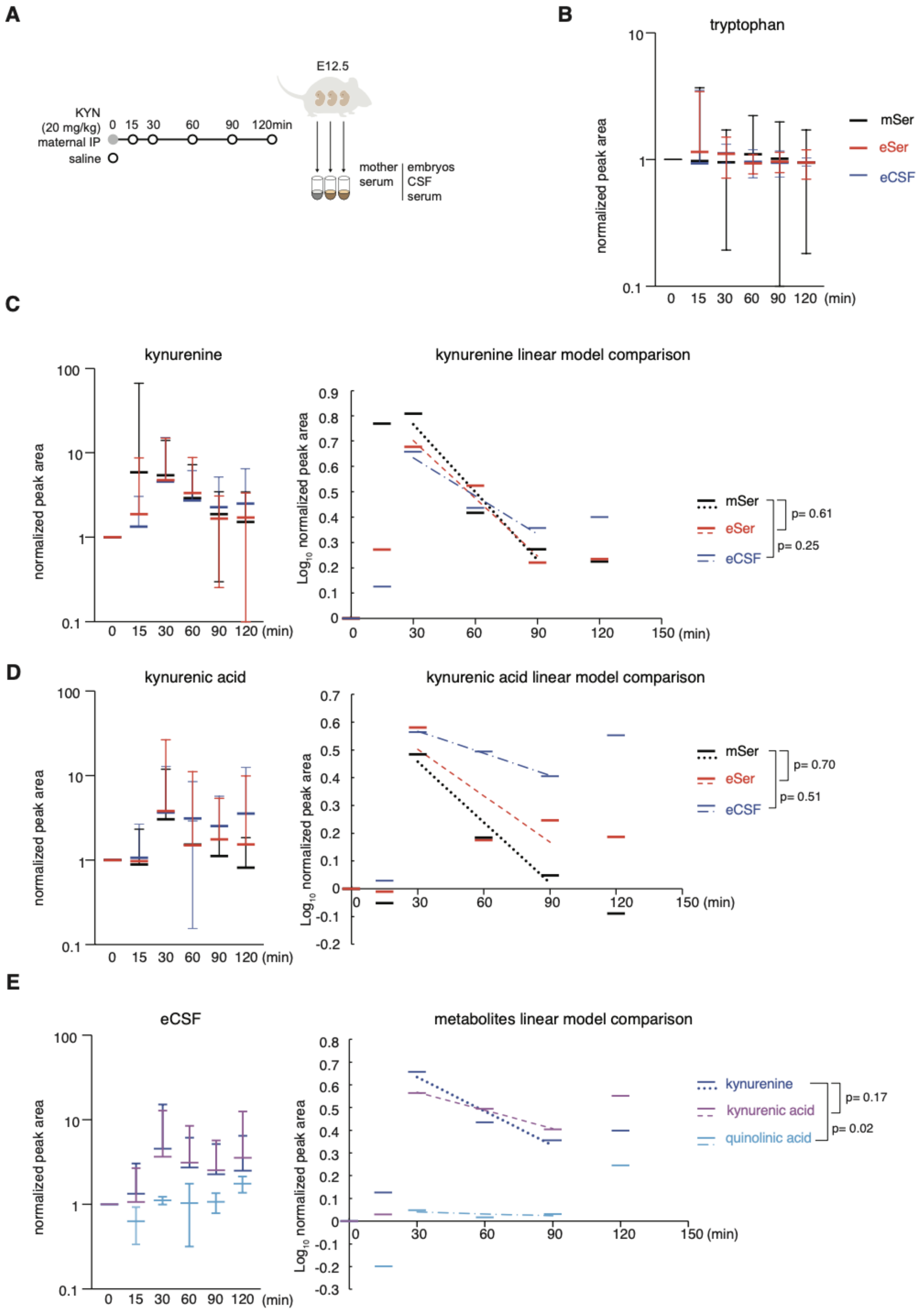

Figure S6. Kynurenine supplementation to the mother results in measurable changes of kynurenine pathway intermediates in the embryo.

A Schematic depicting the IP-injection strategy for kynurenine supplementation. Several time points were collected, time point zero was from mothers injected with saline. eCSF, embryonic serum (eSer), and maternal serum (mSer) were collected for targeted LC-MS metabolomics. At each time point, blood was collected from each mother, and her litter of embryos was harvested and eCSF and eSer were pooled.

B Mean and standard deviation of tryptophan levels in indicated biofluids at different time points following maternal kynurenine supplementation. Each replicate represents an independent pregnant dam or pooled eSer or eCSF from a corresponding litter. n=3, except for the time point 15 minutes, where n=2. Data were normalized to saline-injected control dams.

C, D Left panels: as in B but for kynurenine (C), and kynurenic acid (D). Right panels: corresponding trajectory plots for relative levels of each metabolite in indicated biofluids. Statistics model testing was based on logarithmic decay where slopes of log-transformed data were compared. Individual p-values are depicted. n=3, except for the time point 15 minutes, where n=2.

E As in C and D but relative levels of kynurenine, kynurenic acid, and quinolinic acid in eCSF are compared across different time points post maternal kynurenine supplementation.

Figure S7

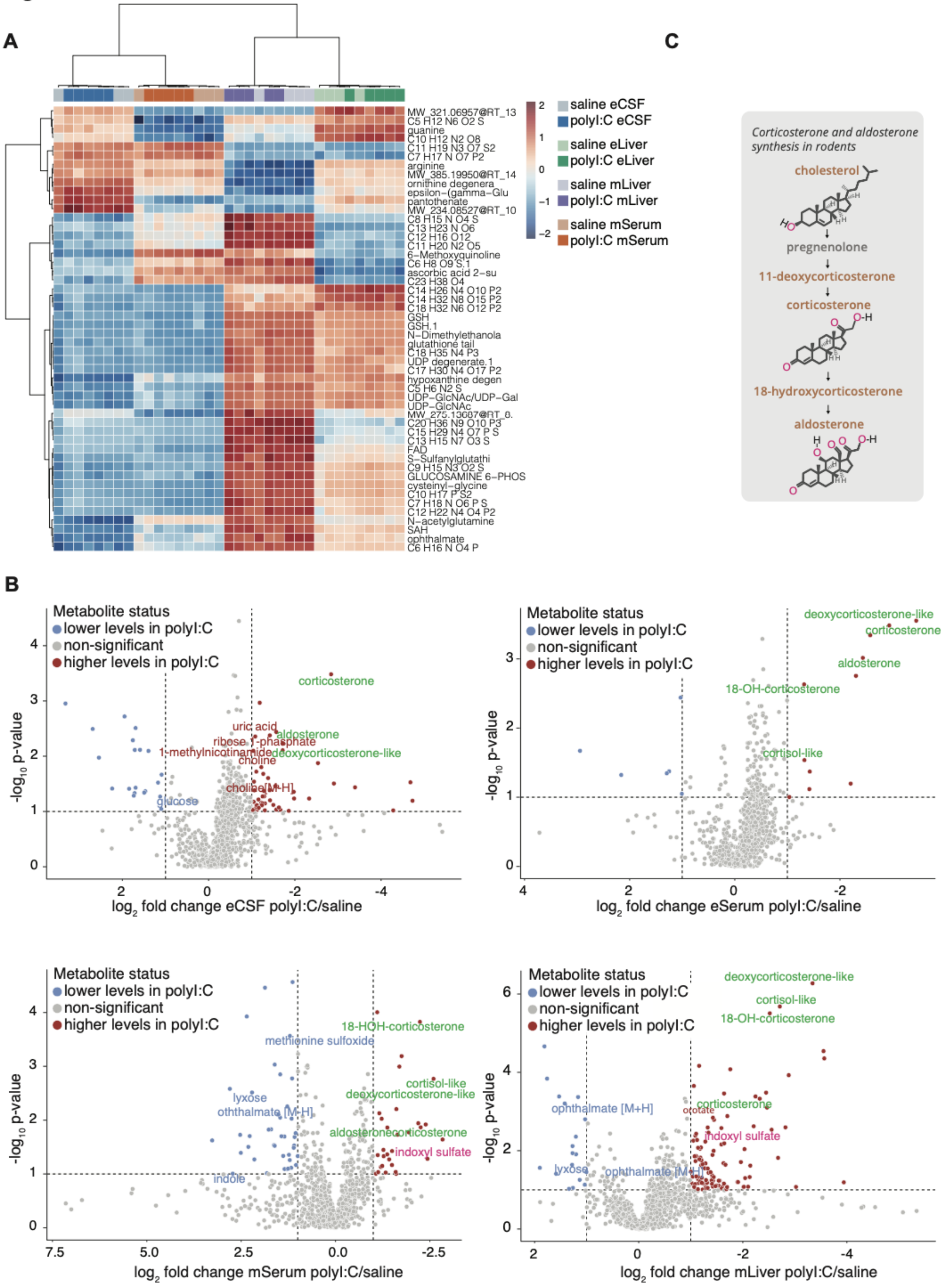

Figure S7. Assessment of metabolic changes in the maternal-fetal axis following MIA induction by untargeted metabolomics.

A Heatmap of the 50 most changed metabolites in a comparison between MIA and control maternal and embryonic tissues including embryonic liver (eLiv), eCSF, maternal liver (mLiv) and maternal serum (mSer) by untargeted metabolomics. Level 1-3 (including matches to the CD-1 database presented in Figure 1) confidence metabolites were used for this analysis. Data was analyzed using the online MetaboAnalyst tool, after log transformation and Pareto scaling of normalized positive and negative-mode combined datasets.

B Volcano plots depicting a comparison between metabolites detected by untargeted metabolomics from eLiv, eCSF, mLiv and mSer following MIA induction by polyI:C delivery. Level 1-3 confidence metabolites were used for this analysis. Significantly changed metabolites are highlighted, only metabolites present in our in-house database are annotated. Blue - lower abundance in polyI:C-treated; red – higher abundance in polyI:C-treated; green – metabolites of interest of the glucocorticoid pathway; and magenta metabolites of interest of the kynurenine pathway. Data were analyzed using the online MetaboAnalyst tool, after log transformation and Pareto scaling of normalized positive and negative-mode combined datasets.

C Schematic of glucocorticoid synthesis in rodents. Metabolites annotated in our untargeted analysis are highlighted in orange.

Figure S8

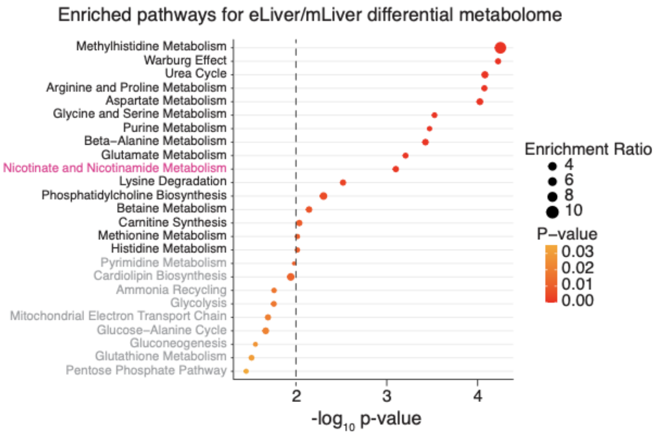

Figure S8. **Assessment of metabolic pathways enrichment in embryonic and maternal livers using untargeted metabolomics.**

Pathway analysis of significantly different metabolites (both lower and higher abundance) between embryonic (eLiver) and maternal (mLiver) liver. Data is from untargeted metabolomics of samples at 3hrs post saline injection (experiment 2; corresponding to E12.5 and presented in Figures 5 and 6 and supplementary Figure S3). Level 1-3 confidence metabolites were used for this analysis. For Level 3 compounds, metabolite annotation corresponding to the first hit to an online database (Metabolika or ChemSpider) was used. Data was analyzed using the online MetaboAnalyst tool, after log transformation and pareto scaling of normalized positive and negative-mode combined datasets.

Supplementary Table S1: **Retention times and chemical formula for compounds in the MS1-level "HILIC_all" database.**

Compound Name	Compound Formula	Retention Time
1,4-BUTANEDIAMINE_13C4	[13]C4H12N2	2.64
1-methylnicotinamide	C7H9N2O	12.02
2,3-BPG_unc	C3H8O10P2	12.7
2-aminobutyric_acid	C4H9NO2	7.96
2-hydroxybutyric_acid	C4H8O3	4.1
2-hydroxyglutarate	C5H8O5	10.2
2-oxoadipate_unc	C6H8O5	9.8
2-oxobutyric_acid_unc	C4H6O3	3.73
3-hydroxybutyric_acid	C4H8O3	4.9
3-hydroxyisobutyric_acid	C4H8O3	5.5
3-methyl-2-oxopentanoic_acid	C6H10O3	2.49
3-phosphoglycerate	C3H7O7P	11.26
3-ureidopropionate	C4H8N2O3	7.85
4-Hydroxyphenylpyruvic acid	C9H8O4	3.97
5,10-CH2-H4PteGlu	C20H24N7O6	10
5,10-CH=H4PteGlu	C20H22N7O6	10.7
5-CH3-H4PteGlu	C20H26N7O6	9.4
5/10-CHO-H4PteGlu	C20H24N7O7	9.84
6-phosphogluconate	C6H13O10P	12.58
acetoacetate	C4H6O3	4.23
Acetyl CoA	C23H38N7O17P3S	8.17
acetylalanine	C5H9NO3	4.7
acetylaspartate	C6H9NO5	9.9
acetylcarnitine	C9H17NO4	5.56
acetylglutamate	C7H11NO5	9.62
acetylglycine	C4H7NO3	5.75
acetylserine	C5H9NO4	6.56
adenine	C5H5N5	4.51
adenosine_unc	C10H13N5O4	4.1
ADP	C10H15N5O10P2	10
ADP-ribose_unc	C15H23N5O14P2	10.47
AICAR	C9H15N4O8P	10.35
alanine	C3H7NO2	9.02
alanine-13C3-15N	[13]C3H7[15]NO2	9.02
allantoin	C4H6N4O3	8.27
alpha-keto-isovalerate	C5H8O3	2.85
Alpha-Ketoglutarate	C5H6O5	13.17

ALPHA-KETOGLUTARIC ACID_1,2,3,4-13C4	[13]C4C1H6O5	10.34
Alpha-Lipoic Acid_unc	C8H14O2S2	2.8
amidoadipate	C6H11NO4	10.06
AMP	C10H14N5O7P	9.3
anserine_unc	C10H16N4O3	8.61
anthranilic_acid	C7H7NO2	4
arginine	C6H14N4O2	14.23
arginine-13C6-15N4	[13]C6H14[15]N4O2	14.23
argininosuccinate_unc	C10H18N4O6	11.47
ascorbate_unc	C6H8O6	9.56
asparagine	C4H8N2O3	9.32
aspartate	C4H7NO4	9.93
aspartate-13C6-15N4	[13]C4H7[15]NO4	9.93
asymmetric dimethylarginine (ADMA)	C8H18N4O2	11.75
ATP	C10H16N5O13P3	10.85
beta-alanine	C3H7NO2	9.45
betaine	C5H11NO2	5.94
biotin	C10H16N2O3S	4.49
Butyrylcarnitine	C11H21NO4	3.54
C5 Carnitines	C12H23NO4	3.26
cAMP_unc	C10H12N5O6P	5.24
carnitine	C7H15NO3	7.68
carnosine	C9H14N4O3	9.44
CDP	C9H15N3O11P2	11.84
CDP-choline	C14H27N4O11P2	10.01
Chenodeoxycholic acid	C24H40O4	2.31
Cholic acid	C24H40O5	2.55
choline	C5H14NO	9.04
cis-aconitate	C6H6O6	12.15
citrate	C6H8O7	12.2
CITRIC ACID_1,5,6-CARBOXYL-13C3	[13]C3C3H8O7	12.15
citrulline	C6H13N3O3	9.73
CMP	C9H14N3O8P	10.6
CoA-Ellmans_unc		10
Coenzyme A	C21H36N7O16P3S	8.9
creatine	C4H9N3O2	8.78
creatinine	C4H7N3O	4.56
CREATININE_N-METHYL-D3	C4H4D3N3O	4.54
CTP	C9H16N3O14P3	13

Cys_Ell		8.2
Cys_Gly_Ell		9
cystathionine	C7H14N2O4S	10.91
cysteic_acid	C3H7NO5S	10.48
cysteine-glycine	C5H10N2O3S	6.6
cysteine_13C3_15N1	[13]C3H7[15]NO2S	3.8
cysteine_unc	C3H7NO2S	3.8
cystine	C6H12N2O4S2	10.4
cystine-13C6-15N2	[13]C6H12[15]N2O4S2	10.4
cytidine	C9H13N3O5	6.4
cytosine_unc	C4H5N3O	5.88
dATP	C10H16N5O12P3	10.25
dCTP	C9H16N3O13P3	11.47
dCytidine	C9H13N3O4	5.15
Deoxycholic acid	C24H40O4	2.31
dihydroorotate_unc	C5H6N2O4	7.33
dihydroxyacetone_phosphate	C3H7O6P	10.44
dimethylglicine	C4H9NO2	6.9
dTMP	C10H15N2O8P	8.54
dTTP	C10H17N2O14P3	11.4
dUMP	C9H13N2O8P	9.64
erythrose_4-phosphate_unc	C4H9O7P	11.35
ETHANOLAMINE_1,1,2,2-D4	C2H3D4NO	10.34
F6P/G1P_unc	C6H13O9P	10.6
FAD	C27H33N9O15P2	7.14
folic_acid	C19H19N7O6	11.4
formyl_methionine	C6H11NO3S	3.3
fructose	C6H12O6	7.86
Fructose 1,6-bisphosphate	C6H14O12P2	12.12
fumarate	C4H4O4	10.64
FUMARIC ACID_13C4	[13]C4H4O4	10.75
gamma-aminobutyric_acid	C4H9NO2	9.43
gamma-glutamyl-alanine	C8H15N3O4	9.53
GAR	C7H15N2O8P	11.21
GDP	C10H15N5O11P2	12.4
glucose	C6H12O6	9.04
Glucose 6-phosphate	C6H13O9P	11.28
glutamate	C5H9NO4	9.78
glutamate-13C5,15N	[13]C5H9[15]NO4	9.78
glutamine	C5H10N2O3	9.15

glyceraldehyde_3-phosphate	C3H7O6P	11.35
glycerate	C3H6O4	7.46
Glycerol 3-Phosphate	C3H9O6P	9.86
Glycerophosphocholine	C8H20NO6P	8.75
glycine	C2H5NO2	9.74
glycine_13C2-15N	[13]C2H5[15]NO2	9.7
GMP	C10H14N5O8P	11.21
GSH	C10H17N3O6S	9.59
GSH-13C2_15N	[13]C2C8H17[15]N1N2O6S	9.6
GSH_13C2-15N-Ellmans	[13]C2C15H21[15]N1N3O10S2	10
GSH_Ellmans	C17H21N4O10S2	10
GSSG	C20H32N6O12S2	11.95
GSSG-13C4_15N2	[13]C4C16H32[15]N2N4O12S2	11.9
GTP	C10H16N5O14P3	12.93
guanidinoacetate	C3H7N3O2	9.6
guanine	C5H5N5O	6.9
guanosine	C10H13N5O5	7.23
GUANOSINE_15N5	C10H13[15]N5O5	7.2
H2PteGlu	C19H22N7O6	10.5
H4PteGlu	C19H24N7O6	10
HIAA	C10H9NO3	8.44
histidine	C6H9N3O2	8.94
histidine-13C6-15N3	[13]C6H9[15]N3O2	8.94
HoCy_Ell		8
homocitrulline	C7H15N3O3	9.4
homocysteic_acid	C4H9NO5S	12.72
homocysteine	C4H9NO2S	7.45
Homocysteine sulfinic acid	C4H9NO4S	9.86
hydroxyproline_gammaALA	C5H9NO3	8.9
hypotaurine	C2H7NO2S	9.12
hypoxanthine	C5H4N4O	5.19
HYPOXANTHINE_13C5	[13]C5H4N4O	5.2
IMP	C10H13N4O8P	10.43
INDOLE-3-ACETIC ACID_PHENYL-13C6	[13]C6C4H9NO2	4.55
indolelactate	C11H11NO3	3.85
inosine	C10H12N4O5	6.05
isocitrate_unc	C6H8O7	14.13
isoleucine	C6H13NO2	5.88
Isoleucine-13C6-15N	[13]C6H13[15]NO2	5.88
Itaconic Acid	C5H6O4	10.1

Kynurenic Acid	C10H7NO3	3.2
kynurenine	C10H12N2O3	5.26
L-ALANINE_13C3	[13]C3H7[15]NO2	8.9
L-LEUCINE_13C6	[13]C6H13NO2	5.3
L-PHENYLALANINE_RING-13C6	[13]C6C3H11NO2	4.8
L-TRYPTOPHAN_13C11	[13]C11H12N2O2	6.15
L-TYROSINE_RING-13C6	[13]C6C3H11NO3	7.5
lactic_acid	C3H6O3	5.46
leucine	C6H13NO2	5.48
leucine-13C6-15N	[13]C6H13[15]NO2	5.48
lysine	C6H14N2O2	13.89
lysine-13C6-15N2	[13]C6H14[15]N2O2	13.89
Lythocholic acid	C24H40O3	2.14
malate	C4H6O5	10.81
malonyl-CoA	C24H38N7O19P3S	10.77
mannose	C6H12O6	8.4
methionine	C5H11NO2S	6.12
Methionine sulfoxide	C5H11NO3S	7.98
methionine-13C5-15N	[13]C5H11[15]NO2S	6.1
methylthioadenosine	C11H15N5O3S	2.72
MTX	C20H22N8O5	10.08
myo-inositol	C6H12O6	10.8
N-acetylcysteine	C5H9NO3S	4.3
N-acetylglutamate	C7H11NO5	9.93
N-acetylglutamine	C7H12N2O4	6.29
N-acetyllysine	C8H16N2O3	7.58
N-acetylmethionine	C7H13NO3S	3
N-acetylornithine	C7H14N2O3	9.65
N-acetylphenylalanine	C11H13NO3	2.34
NAD+	C21H27N7O14P2	9.34
NADH	C21H29N7O14P2	8.85
NADP	C21H29N7O17P3	11.14
NADPH	C21H30N7O17P3	11.44
niacinamide	C6H6N2O	3.03
NICOTINAMIDE_13C6	[13]C6H6N2O	3.35
Nicotinic Acid	C6H5NO2	4.2
O-phosphoethanolamine_unc	C2H8NO4P	10.67
ornithine	C5H12N2O2	12.58
orotate	C5H4N2O4	6.1
p-aminobenzoate	C7H7NO2	6.29

palmitate	C16H32O2	2.05
Pantothenate	C9H17NO5	5.3
Phenylacetyl glutamine	C13H16N2O4	3
phenylalanine	C9H11NO2	4.7
phenylalanine-13C9-15N	[13]C9H11[15]NO2	4.7
phosphocholine	C5H14NO4P	10.17
phosphocreatine	C4H10N3O5P	9.85
phosphoenolpyruvate	C3H5O6P	11.8
phosphoethanolamine	C2H8NO4P	11
phosphoserine	C3H8NO6P	11.28
Picolinate	C6H4NO2	4.36
Pipecolate	C6H11NO2	6.8
proline	C5H9NO2	7.23
proline-13C5-15N	[13]C5H9[15]NO2	7.28
propionylcarnitine	C10H19NO4	4.74
PRPP_unc	C5H13O14P3	13.21
pseudouridine_unc	C9H12N2O6	6.91
PteGlu	C19H20N7O6	11.33
Pyridoxal	C8H9NO3	3.76
Pyridoxine	C8H11NO3	3.64
pyruvate	C3H4O3	4.3
PYRUVATE_13C3	[13]C3H4O3	4.35
quinolinic_acid	C7H5NO4	10.38
Riboflavin	C17H20N4O6	3.49
Ribose 1-phosphate_unc	C5H11O8P	10.37
Ribose/Ribulose 5-phosphate/3Me-Lys	C5H11O8P	10.54
S-Adenosyl-L-Homocysteine	C14H20N6O5S	7.8
S-Adenosyl-L-methionine	C15H22N6O5S	9.94
S-methylcysteine_unc	C4H9NO2S	6.69
saccharopine_unc	C11H20N2O6	10.54
sarcosine_unc	C3H7NO2	8.48
Sedoheptulose-7-phosphate	C7H15O10P	11.04
serine	C3H7NO3	9.59
serine-13C3-15N	[13]C3H7[15]NO3	9.59
serotonin	C10H12N2O	14.04
SODIUM PALMITATE_U-13C16	[13]C16H32O2	2.05
sorbitol	C6H14O6	8.2
succinate	C4H6O4	10.19
succinyl-CoA	C7H13NO3S	10.34
Succinylacetone	C7H10O4	3.36

taurine	C2H7NO3S	9.26
thiamine	C12H16N4OS	6.64
threonine	C4H9NO3	8.61
threonine-13C4-15N	[13]C4H9[15]NO3	8.61
thymidine	C10H14N2O5	3.06
THYMINE_1,3-15N2	C5H6[15]N2O2	5.1
trans-urocanate	C6H6N2O2	6.53
tryptamine	C10H12N2	11.4
tryptophan	C11H12N2O2	6.16
tyrosine	C9H11NO3	7.58
tyrosine-13C9-15N	[13]C9H11[15]NO3	7.58
UDP	C9H14N2O12P2	11.09
UDP-GlcNAc	C17H27N3O17P2	10.1
UDP-hexose	C15H24N2O17P2	11.08
UMP	C9H13N2O9P	10.15
uracil	C4H4N2O2	4.15
Ureidosuccinic_acid	C5H8N2O5	11.24
uric acid	C5H4N4O3	7.97
uridine	C9H12N2O6	4.81
UTP	C9H15N2O15P3	12.13
valine	C5H11NO2	7
valine-13C5-15N	[13]C5H11[15]NO2	7.01
xanthine	C5H4N4O2	7.15
xanthosine_unc	C10H12N4O6	7.8

Supplementary Table S2. Retention times and chemical formula for compounds in the MS^1-level "MSMLS_HILIC" database.

Compound Name	Compound Formula	Retention Time
sorbose	C6H12O6	8.47
xylitol	C5H12O5	7.74
ribitol	C5H12O5	7.7
myoinositol	C6H12O6	10.01
mannose	C6H12O6	8.57
xylose	C5H10O5	7.96
sucrose	C12H22O11	9.5
galactose	C6H12O6	9.3
alpha-d-glucose	C6H12O6	9.21
allose	C6H12O6	8.68
mannitol	C6H14O6	8.63
melibiose	C12H22O11	10.61
sorbitol	C6H14O6	8.54
maltose	C12H22O11	10.05
tagatose	C6H12O6	8.06
l-gulonolactone	C6H10O6	7.49
arabinose	C5H10O5	7.9
cellobiose	C12H22O11	10.06
psicose	C6H12O6	7.09
arabitol	C5H12O5	7.75
lyxose	C5H10O5	7.58
ribose	C5H10O5	6.66
palatinose	C12H22O11	9.59
d-pinitol	C7H14O6	7.99
putrescine	C4H12N2	
deoxycarnitine	C7H15NO2	8.05
adenosine 2',3'-cyclic phosphate	C10H12N5O6P	4.79
mevalolactone	C6H10O3	2.39
galactose 1-phosphate	C6H13O9P	10.8
dimethylallylpyrophosphate	C5H12O7P2	8.38
deoxyuridine triphosphate	C9H15N2O14P3	11.38
dgdp	C10H15N5O10P2	11.42
5-methylcytosine	C5H7N3O	5.05
glycerate	C3H6O4	7.78
cytidine 2',3'-cyclic phosphate	C9H12N3O7P	8.07
n,n,n-trimethyllysine	C9H20N2O2	12.5
riboflavin	C17H20N4O6	3.71

uridine diphosphate glucose	C15H24N2O17P2	11.21
methyl galactoside	C7H14O6	6.31
pyridoxal-phosphate	C8H10NO6P	9.66
dihydroxyacetone phosphate	C3H7O6P	10.4
phosphoenolpyruvate	C3H5O6P	11.99
mannose 6-phosphate	C6H13O9P	10.99
3-phosphoglycerate	C3H7O7P	11.62
l-carnitine	C7H15NO3	7.97
o-phosphoethanolamine	C2H8NO4P	10.66
cytidine monophosphate	C9H14N3O8P	10.76
guanosine diphosphate mannose	C16H25N5O16P2	12.54
adp-glucose	C16H25N5O15P2	10.08
fructose 6-phosphate	C6H13O9P	10.86
adenosine 3',5'-diphosphate	C10H15N5O10P2	11.18
3-nitro-l-tyrosine	C9H10N2O5	9.69
p-octopamine	C8H11NO2	8.45
n-alpha-acetyllysine	C8H16N2O3	9.29
uridine diphosphategalactose	C15H24N2O17P2	11.26
pyridoxamine	C8H12N2O2	5.48
5-aminolevulinate	C5H9NO3	8.73
deoxyuridine-monophosphate	C9H13N2O8P	9.34
5'-deoxyadenosine	C10H13N5O3	3.19
ribose 1,5-bisphosphate	C5H12O11P2	12.5
xanthosine-monophosphate	C10H13N4O9P	12.36
fad	C27H33N9O15P2	7.53
orotate	C5H4N2O4	6.44
lauroylcarnitine	C19H38NO4	2.09
n-acetylmethionine	C7H13NO3S	3.14
aicar	C9H15N4O8P	10.24
uridine diphosphate-n-acetylgalactosamine	C17H27N3O17P2	10.41
cyclic gmp	C10H12N5O7P	8.55
homocysteine thiolactone	C4H7NOS	2.73
o-phosphoserine	C3H8NO6P	11.18
s-adenosylhomocysteine	C14H20N6O5S	8.13
l-ornithine	C5H12N2O2	12.75
adenine	C5H5N5	4.52
normetanephrine	C9H13NO3	7.25
uridine diphosphate-n-acetylglucosamine	C17H27N3O17P2	10.34
guanosine diphosphate	C10H15N5O11P2	12.21
glutathione reduced	C10H17N3O6S	10.32

uridine diphosphate glucuronic acid	C15H22N2O18P2	13.25
n,n-dimethylarginine	C8H18N4O2	11.77
cytidine diphosphate	C9H15N3O11P2	11.64
selenocystamine	C4H12N2Se2	13.7
histamine	C5H9N3	12.35
indoxyl sulfate	C8H7NO4S	4.15
thiamine monophosphate	C12H17N4O4PS	9.09
uracil 5-carboxylate	C5H4N2O4	9.14
glyoxylate	C2H2O3	11
guanosine monophosphate	C10H14N5O8P	11.36
n-acetylalanine	C5H9NO3	5.11
4-guanidinobutanoate	C5H11N3O2	9.32
d-mannosamine	C6H13NO5	8.71
diaminopimelate	C7H14N2O4	11.63
aminoadipate	C6H11NO4	10.02
deoxycytidine	C9H13N3O4	5.49
glucosamine 6-phosphate	C6H14NO8P	10.9
tartrate	C4H6O6	11.66
3-dehydroshikimate	C7H8O5	7.78
caffeine	C8H10N4O2	2.29
homocysteine	C4H9NO2S	7.76
theophylline	C7H8N4O2	2.68
leucine	C6H13NO2	5.78
trehalose	C12H22O11	10.19
betaine	C5H11NO2	6.33
tryptophan	C11H12N2O2	6.54
3-sulfinoalanine	C3H7NO4S	9.81
o-succinyl-homoserine	C8H13NO6	8.94
allantoin	C4H6N4O3	8.53
d-glucuronolactone	C6H8O6	5.59
(2-aminoethyl)phosphonate	C2H8NO3P	10.7
2,5-dihydrobenzoic acid	C7H6O4	5.27
maleimide	C4H3NO2	6.7
threitol	C4H10O4	6.75
paraxanthine	C7H8N4O2	2.7
adenosine 5'-diphosphate	C10H15N5O10P2	10.11
2-deoxy-d-glucose	C6H12O5	8.74
1-methyl-l-histidine	C7H11N3O2	7.24
galactitol	C6H14O6	8.74
oxoproline	C5H7NO3	6.4

4-pyridoxate	C8H9NO4	2.31
quinolinate	C7H5NO4	10.26
methylguanidine	C2H7N3	19.3
deoxyguanosine-monophosphate	C10H14N5O7P	10.48
3-hydroxy-3-methylglutaryl-coa	C27H44N7O20P3 S	9.96
glucuronate	C6H10O7	10.8
1-methyladenosine	C11H15N5O4	6.78
deoxyuridine	C9H12N2O5	3.7
gluconate	C6H12O7	9.37
urocanate	C6H6N2O2	6.83
kynurenine	C10H12N2O3	5.77
pyroglutamate	C5H7NO3	6.39
4-acetamidobutanoate	C6H11NO3	5.05
adenosine-monophosphate	C10H14N5O7P	9.16
lysine	C6H14N2O2	13.99
phosphoserine	C3H8NO6P	11.1
1-aminocyclopropanecarboxylate	C4H7NO2	7.49
cystathionine	C7H14N2O4S	10.87
norvaline	C5H11NO2	6.95
3-hydroxymethylglutarate	C6H10O5	9.55
phosphonoacetate	C2H5O5P	11.13
picolinate	C6H5NO2	4.57
arginine	C6H14N4O2	14.53
trans-4-hydroxy-l-proline	C5H9NO3	9.04
homocystine	C8H16N2O4S2	10.19
n-methylglutamate	C6H11NO4	8.48
d-ornithine	C5H12N2O2	12.83
xanthosine	C10H12N4O6	7.93
3-methylcrotonyl-coa	C26H42N7O17P3 S	6.56
thyrotropin releasing hormone	C16H22N6O4	4.18
cysteate	C3H7NO5S	10.66
n-methylaspartate	C5H9NO4	8.74
galactarate	C6H10O8	11.72
alpha-hydroxyisobutyrate	C4H8O3	4.3
nicotinic acid adenine dinucleotide phosphate ((naadp))	C21H27N6O18P3	11.76
n-acetylasparagine	C6H10N2O4	7.38
pipecolate	C6H11NO2	7.18
glucose 6-phosphate	C6H13O9P	11.41
nadp	C21H28N7O17P3	11.27

6-phosphogluconate	C6H13O10P	12.05
isopentenyl pyrophosphate	C5H12O7P2	8.34
guanosine triphosphate	C10H16N5O14P3	12.96
dtdp-d-glucose	C16H26N2O16P2	9.62
dgtp	C10H16N5O13P3	12.15
n-acetylglycine	C4H7NO3	6.2
n-acetylaspartate	C6H9NO5	9.9
inosine 5'-diphosphate	C10H14N4O11P2	11.34
nicotinamide hypoxanthine dinucleotide	C21H26N6O15P2	10.41
s-adenosylmethionine	C15H23N6O5S	9.87
erythritol	C4H10O4	6.72
glucosaminate	C6H13NO6	10.84
uridine triphosphate	C9H15N2O15P3	12.04
2-keto-3-deoxy-d-gluconic acid	C6H10O6	7.84
d-sedoheptulose	C7H14O7	8.64
deoxyadenosine	C10H13N5O3	3.41
n-acetylputrescine	C6H14N2O	10.6
n-acetylgalactosamine	C8H15NO6	6.67
n-acetylglutamate	C7H11NO5	9.47
6-hydroxynicotinate	C6H5NO3	6.74
inosine-monophosphate	C10H13N4O8P	10.47
pantothenate	C9H17NO5	4.52
aniline-2-sulfonate	C6H7NO3S	4.39
s-carboxymethylcysteine	C5H9NO4S	9.73
thiamine pyrophosphate	C12H18N4O7P2S	9.55
histidinol	C6H11N3O	8.94
thymidine-monophosphate	C10H15N2O8P	8.6
ureidopropionate	C4H8N2O3	7.72
5-aminopentanoate	C5H11NO2	9.58
norleucine	C6H13NO2	5.54
n-formylglycine	C3H5NO3	6.87
adenosine	C10H13N5O4	4.05
raffinose	C18H32O16	10.67
meso-tartrate	C4H606	11.08
saccharate	C6H10O8	11.61
adenosine triphosphate	C10H16N5O13P3	10.93
3-methoxytyrosine	C10H13NO4	6.81
lactose	C12H22O11	10.29
4-imidazoleacetate	C5H6N2O2	7.23
galacturonate	C6H10O7	11.02

cytidine triphosphate	C9H16N3O14P3	12.31
cyclic amp	C10H12N5O6P	5.42
methionine sulfoximine	C5H12N2O3S	8.62
cis-4-hydroxy-d-proline	C5H9NO3	9.22
glucosamine 6-sulfate	C6H13NO8S	11.21
nadph	C21H30N7O17P3	10.99
3-methylhistamine	C6H11N3	8.33
maleamate	C4H5NO3	6.73
methyl 4-aminobutyrate	C5H11NO2	7.59
n-formyl-l-methionine	C6H11NO3S	3.34
oxalate	C2H2O4	12.04
5-hydroxytryptophan	C11H12N2O3	8.85
d-alanine	C3H7NO2	9.03
theobromine	C7H8N4O2	2.77
guanidinosuccinate	C5H9N3O4	10.32
histidine	C6H9N3O2	8.81
allothreonine	C4H9NO3	8.73
phosphocreatine	C4H10N3O5P	10.19
adenosine diphosphate ribose	C15H23N5O14P2	9.9
citramalate	C5H8O5	9.92
anserine	C10H16N4O3	8.43
biliverdin	C33H34N4O6	1.95
cysteamine	C2H7NS	8.61
ophthalmate	C11H19N3O6	8.84
mesoxalate	C3H2O5	13.18
trigonelline	C7H7NO2	6.53
3,4-dihydroxyphenylglycol	C8H10O4	5.11
2-hydroxybutyrate	C4H8O3	4.18
coenzyme a	C21H36N7O16P3 S	8.98
inosine triphosphate	C10H15N4O14P3	12.21
cdp-ethanolamine	C11H20N4O11P2	10.92
stachyose	C24H42O21	11.67
deoxycytidine-diphosphate	C9H15N3O10P2	10.78
hydroxykynurenine	C10H12N2O4	7.52
galactosamine	C6H13NO5	8.98
deoxyadenosine triphosphate	C10H16N5O12P3	10.33
glycerol 3-phosphate	C3H9O6P	10.02
4-hydroxy-l-phenylglycine	C8H9NO3	9.3
n-acetylserine	C5H9NO4	6.73

uridine 5'-diphosphate	$C_9H_{14}N_2O_{12}P_2$	11.12
